# Lymphatic-preserving treatment sequencing with immune checkpoint inhibition unleashes cDC1-dependent antitumor immunity in HNSCC

**DOI:** 10.1038/s41467-022-31941-w

**Published:** 2022-07-25

**Authors:** Robert Saddawi-Konefka, Aoife O’Farrell, Farhoud Faraji, Lauren Clubb, Michael M. Allevato, Shawn M. Jensen, Bryan S. Yung, Zhiyong Wang, Victoria H. Wu, Nana-Ama Anang, Riyam Al Msari, Shiruyeh Schokrpur, Ida Franiak Pietryga, Alfredo A. Molinolo, Jill P. Mesirov, Aaron B. Simon, Bernard A. Fox, Jack D. Bui, Andrew Sharabi, Ezra E. W. Cohen, Joseph A. Califano, J. Silvio Gutkind

**Affiliations:** 1 Department of Otolaryngology-Head and Neck Surgery, UC San Diego School of Medicine, San Diego, CA USA; 2 Moores Cancer Center, UC San Diego, La Jolla, CA USA; 3 Gleiberman Head and Neck Cancer Center, UC San Diego, La Jolla, CA USA; 4 Department of Bioengineering, University of Pennsylvania, Philadelphia, PA USA; 5 Earle A Chiles Research Institute, Robert W Franz Cancer Research Center, Providence Portland Medical Center, Portland, OR USA; 6 Department of Medicine, Division of Hematology-Oncology, UC San Diego School of Medicine, San Diego, CA USA; 7 Department of Medicine, UC San Diego School of Medicine, La Jolla, CA USA; 8 Department of Radiation Oncology, UC Irvine School of Medicine, Irvine, CA USA; 9 Department of Molecular Microbiology and Immunology, Oregon Health Science University, Portland, OR USA; 10 Department of Pathology, UC San Diego School of Medicine, La Jolla, CA USA; 11 Department of Radiation Medicine and Applied Sciences, UC San Diego School of Medicine, San Diego, CA USA; 12 Department of Pharmacology, UC San Diego, La Jolla, CA USA

**Keywords:** Cancer immunotherapy, Head and neck cancer, Cancer models, Tumour immunology

## Abstract

Despite the promise of immune checkpoint inhibition (ICI), therapeutic responses remain limited. This raises the possibility that standard of care treatments delivered in concert may compromise the tumor response. To address this, we employ tobacco-signature head and neck squamous cell carcinoma murine models in which we map tumor-draining lymphatics and develop models for regional lymphablation with surgery or radiation. We find that lymphablation eliminates the tumor ICI response, worsening overall survival and repolarizing the tumor- and peripheral-immune compartments. Mechanistically, within tumor-draining lymphatics, we observe an upregulation of conventional type I dendritic cells and type I interferon signaling and show that both are necessary for the ICI response and lost with lymphablation. Ultimately, we provide a mechanistic understanding of how standard oncologic therapies targeting regional lymphatics impact the tumor response to immune-oncology therapy in order to define rational, lymphatic-preserving treatment sequences that mobilize systemic antitumor immunity, achieve optimal tumor responses, control regional metastatic disease, and confer durable antitumor immunity.

## Abstract (web summary)

Response rates to immune checkpoint inhibitors (ICI) in patients with head and neck squamous cell carcinoma (HNSCC) remain low. Here the authors show that ablative treatment of tumor-draining regional lymphatics, a standard of care approach in patients, impairs the tumor response to ICI in preclinical HNSCC models.

## Introduction

Worldwide, head and neck squamous cell carcinoma (HNSCC) represents a significant health issue, with more than 600,000 new cases diagnosed each year and approximately one-half of all patients succumbing to their disease^1–3^. Arising from the upper aerodigestive tract, HNSCCs include cancers of the oral cavity, oropharynx, larynx, and hypopharynx. Most often, HNSCCs are diagnosed at late stages (stages III–IV) with roughly 60% of patients harboring locally advanced disease at the time of presentation^2,3^. Historically, definitive-intent treatment for HNSCC patients included surgery and radiotherapy, which incur significant treatment-associated morbidity and have resulted in modest improvements in rates of cure. Subsequent efforts to improve outcomes included the addition of molecularly targeted therapies,^4,5^ such as the EGFR-blocking antibody Cetuximab^6^, and cisplatin chemotherapy to radiation^7–11^. Even with these advances, including efforts to minimize treatment-related toxicity^12–14^, the 5-year overall survival in patients with late-stage disease approaches only 50%, and locoregional and distant recurrence rates remain high^2,3,15^.

Immune checkpoint inhibitor (ICI) therapy offers the potential to improve oncologic outcomes for patients with HNSCC while reducing treatment-associated morbidity. Targeting either programmed cell death protein (PD-1), the cognate ligand (PD-L1) or cytotoxic T lymphocyte-associated protein 4 (CTLA-4), ICIs invigorate endogenous antitumor immunity by releasing peripheral inhibition on antitumor T cells^16^. The introduction of ICIs into clinical practice has revolutionized the treatment of several malignancies^17^ and changed the paradigm for the treatment of recurrent/metastatic (r/m) HNSCC. Specifically, the CHECKMATE-141^18^, KEYNOTE-040^19^, and KEYNOTE-048^20^ clinical trials demonstrated an improvement in overall survival for patients with r/m HNSCC treated with αPD-1 versus standard therapies, leading to the approval of αPD-1 ICI in this setting.

While these landmark trials demonstrated a clear benefit for some patients with r/m HNSCC, ICIs have largely underperformed initial expectations and overall objective response rates remain limited with less than 20% of patients showing clinical benefit. Surprisingly, αCTLA-4 ICI, which has demonstrated efficacy in other solid cancers bearing similar mutational burdens^21^ and immune infiltration^22^, has failed to demonstrate benefit for HNSCC patients^23,24^. Moreover, in the curative-intent setting for locally advanced disease, emerging evidence now indicates that adding αPD-1 ICI to standard radiation with concurrent chemotherapy confers no additional benefit in either progression-free or overall survival^25^. Collectively, these findings raise the possibility that standard of care oncologic therapies for HNSCC may compromise host immunity and the ability to respond to ICI therapy. Given the propensity of HNSCC to harbor occult, regional lymphatic metastasis, elective ablation of cervical lymphatics has become a cornerstone of therapy^26^; and, standard of care management for HNSCC patients with regional metastatic disease necessarily entails either primary surgical or radiation therapy for both involved and uninvolved draining lymph nodes^2,27^. Accordingly, we noted that the landmark clinical trials defining how we currently employ IO therapy in HNSCC necessarily recruited patients who have contemporaneously or previously received ablative locoregional therapies, which prompted us to explore whether this may negatively affect host immunity and the tumor response to ICI. Specifically, we hypothesize that ablative treatment of tumor-draining regional lymphatics may impair the primary tumor response to ICI therapy. We explore this hypothesis using syngeneic animal models of HNSCC in order to develop a rational approach for the effective use of IO therapies in HNSCC. Our results may inform the clinical exploration of new treatment sequencing strategies capable of achieving durable clinical responses.

## Results

### Mapping regional draining lymphatics from the murine head and neck

**Fig. 1:**
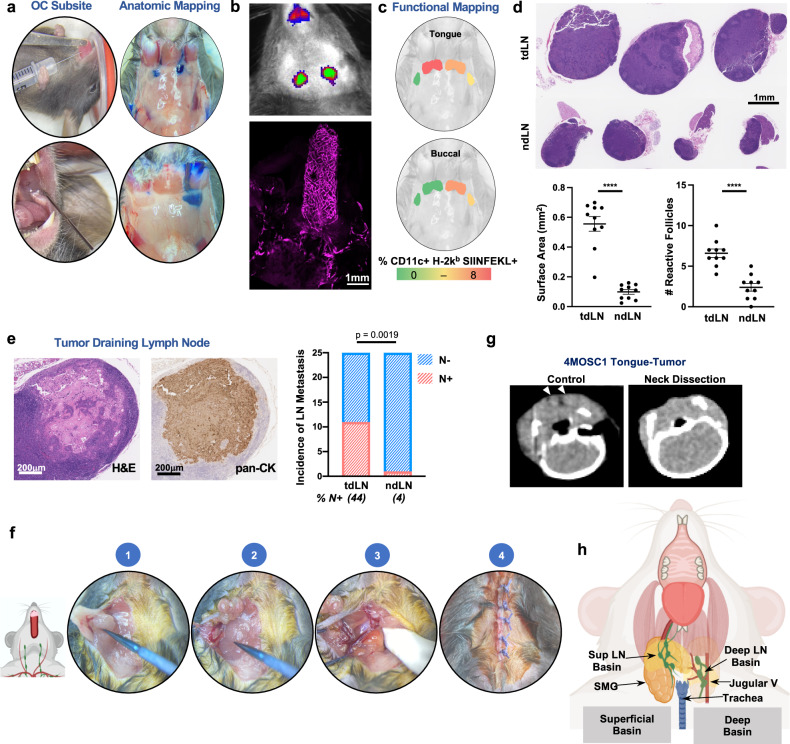
Cervical lymphatic mapping and neck dissection model. **a** Illustrative photographs depicting anatomic lymphatic mapping following injection of 5% Evans Blue dye into oral cavity (OC) subsites. **b** Top: Illustrative IVIS image to depict anatomic lymphatic mapping following injection of anti-LYVE-ef660 antibody into the tongue (1s exposure with the Cy5.5 channel on the IVIS 2000). Bottom: Representative image of a clearing-enhanced 3D (Ce3D) en bloc resected tongue-neck specimen stained with anti-LYVE-ef660 (1:100), imaged with the Leica SP8 confocal microscope. **c** Representative images demonstrating functional mapping following injection of SIINFEKL peptide/CpG adjuvant. Depicted are lymphatic basins, overlayed with heatmaps, to indicate the % CD11c + H-2k^b^ SIINFEKL + cells identified by flow cytometry. **d** Putative tumor-draining lymph nodes (tdLNs) and non-draining lymph nodes (ndLNs) were harvested from 4MOSC1 tumor-bearing animals on day 10. Top: Representative H&E-stained tdLN and ndLN shown with (bottom) scoring for overall surface area and the number of reactive follicles (*n* = 10 independent samples/group). **e** Left: Representative tdLN with the focus of metastatic disease shown, stained by H&E and with anti-pan-CK antibody. Right: Quantification of the incidence of metastatic disease in tdLN and ndLN, shown in a contingency plot (*n* = 25/group, Fisher’s exact test). **f** Illustrative photographs demonstrating the key procedural steps of the murine neck dissection. (1) dissection to reflect the submandibular gland from the superficial lymphatic basin, (2) superficial lymphatic basin liberated from underlying tissues, (3) completed dissection of the deep lymphatic basin with the jugular venous plexus in situ along the floor of the neck, (4) closure. **g** Representative axial CT images of the neck obtained from an untreated 4MOSC1-tongue tumor-bearing animal or following neck dissection (arrowhead = cervical lymph node). **h** Cartoon image to diagram murine cervical lymphatic basins in the context of adjacent, critical head, and neck anatomy. The differences between experimental groups were analyzed using independent, two-sided Student *t* tests (**d**) or fisher’s exact test (**e**). All data represent averages ± SEM, except where indicated. ****P < 0.0001. ns not statistically significant. Source data are provided as a Source Data file.

To explore the contribution of the tumor-draining lymph node (tdLN) in the host response to therapy, we began by anatomically and functionally mapping the murine head and neck (HN) regional lymphatic basins. We delivered Evans Blue dye locally to the oral cavity—tongue and buccal mucosa (Fig. 1a) and retroauricular subcutaneous space (Supplementary Fig. 1A–E). While lymphatics from the tongue drain bilaterally to paired lymph node basins, the buccal space drains only to the ipsilateral lymphatic basin with both oral cavity subsites sparing the deep cervical lymphatic basins, which associate with the internal jugular vein along the floor of the neck (Fig. 1a). To determine whether our anatomic mapping faithfully identifies lymphatic tissues, we performed in vivo imaging following injection of LYVE-ef660, which identifies lymphatic endothelial cells. Following tongue injections, we found that the LYVE-ef660 fluorescence signal overlaps with our anatomic mapping (Fig. 1b, top). Clearing-enhanced three-dimensional imaging (Ce3D)^28^ with LYVE-ef660 revealed the dense arborization of lymphatic channels in the tongue that course into the neck (Fig. 1b, bottom and Supplementary Fig. 1F). To confirm that our anatomic mapping of murine cervical lymphatics reflects functionally relevant, physiologic routes of tumor immunosurveillance, we delivered the model ovalbumin antigen, SIINFEKL, with CpG adjuvant into HN subsites and then probed for lymph node resident antigen-presenting cells (APCs) cross-presenting SIINFEKL peptide by flow cytometry (Fig. 1c and Supplementary Fig. 1G, H). We detected H-2kb-SIINFEKL + APCs in bilateral or ipsilateral LN stations following tongue or buccal injections, respectively (Fig. 1c).

Historically, clinical LN stations have been defined by mapping patterns of metastatic spread in patients with HNSCCs arising from distinct subsites^29^. In a similar fashion, we examined cervical lymphatic basins after establishing orthotopic tumors in the tongue of WT recipient animals (Fig. 1d). To accomplish this, we employed our recently characterized, murine oral squamous cell carcinoma cell line, 4MOSC—a carcinogen-induced, syngeneic model, featuring a human tobacco-signature mutanome and immune infiltrate and ICI response pattern similar to that observed clinically^5,30^. In 4MOSC1-tongue tumor-bearing animals, we found that our a priori mapped draining lymphatics display features consistent with acute immune reactivity: increased cellularity and volume as well as an expansion of secondary follicles (Fig. 1d, bottom). Moreover, we find metastatic spread in putative draining versus non-draining lymphatics disease (11/25 versus 1/25, *P* = 0.0019, respectively; Fig. 1e). Based upon these observations, we define murine, cervical tumor-draining lymphatics (tdLN) as follows: tongue tumors drain to bilateral superficial lymphatic stations while tumors in the buccal space drain to ipsilateral superficial lymphatic basins (see below, Fig. 1h).

### Murine neck dissection model

Mapping the murine cervical lymphatic system allowed us to accurately model lymphatic ablative therapies. Neck dissection to eradicate cervical lymphatics is a cornerstone of contemporary oncologic therapy for HNSCC patients, particularly for oral cavity SCC patients with regional metastatic spread^26,27,31^. To model this preclinically, we developed a neck dissection surgery in the mouse, informed by our cervical lymphatic mapping (Fig. 1f). Following bilateral neck dissection, we observed that Evans Blue injected into the tongue distributes diffusely into the neck (Supplementary Fig. 1I). Post-operative CT scan confirmed the absence of lymph nodes following neck dissection (Fig. 1g).

### Role of tumor-draining lymphatics in the response to ICI in HNSCC

**Fig. 2:**
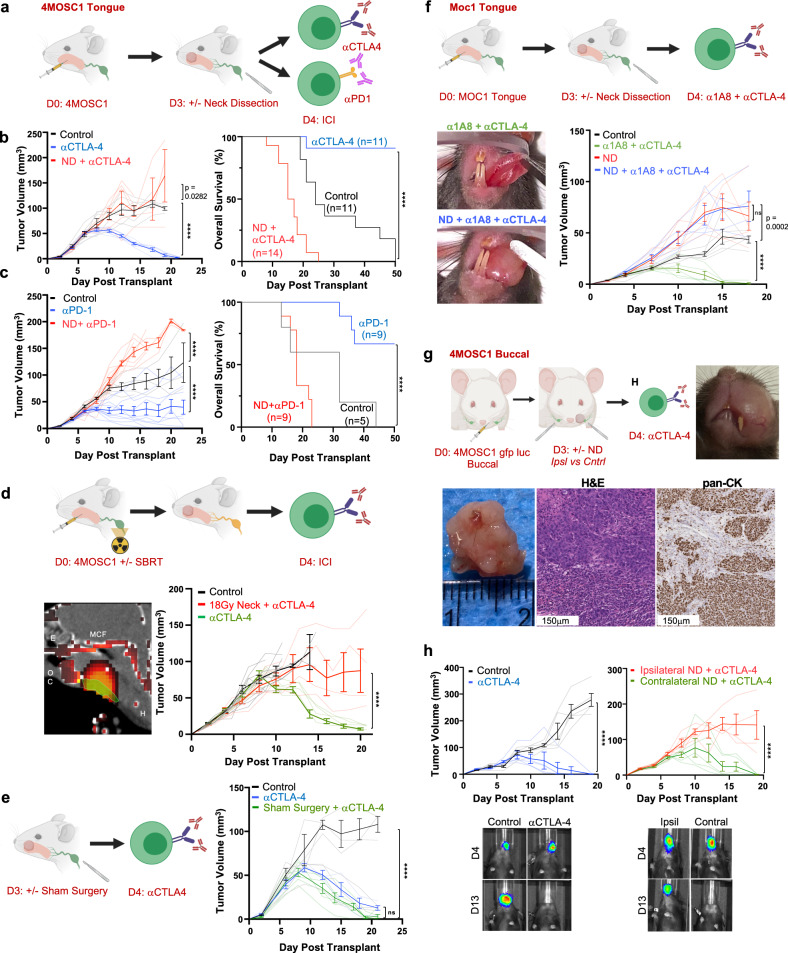
Draining lymphatic basins are required for tumor response to immune checkpoint inhibition. **a** Experimental schema for **b**, **c**. **b** Left: Representative tumor growth kinetics from 4MOSC1 tumor-bearing animals treated with αCTLA-4 following neck dissection (ND, red lines) or no surgery (blue lines) (*n* = 6/group); right: compiled overall survival data (ND + αCTLA-4 ICI, red lines *n* = 14; αCTLA-4 monotherapy, blue lines *n* = 1; control, black lines *n* = 11). **c** Left: Representative tumor growth kinetics from 4MOSC1 tumor-bearing animals treated with αPD-1 following ND (red lines) or no surgery (blue lines) (*n* = 9/treatment group, *n* = 5 control); right: compiled overall survival data (ND + αPD-1 ICI, red lines *n* = 9; αPD-1 monotherapy, blue lines *n* = 9; control, black lines *n* = 5). **d** Top: Experimental schema; bottom left: representative sagittal CT image illustrating a single 18 Gy dose of radiation therapy targeting the cervical lymphatics (green indicates the area of contouring; red heatmap identifies the intensity of delivered ablative single fraction radiation therapy, OC = oral cavity, E = ethmoid, MCF = middle cranial fossa, H = hyoid). Bottom right: Representative tumor growth kinetics from 4MOSC1 tumor-bearing animals treated with αCTLA-4 monotherapy following 18 Gy delivered to the neck on day 0 (red lines, *n* = 5) or no radiation (green lines, *n* = 5) or control animals (black lines, *n* = 6). **e** Left: Experimental schema; right: tumor growth kinetics from 4MOSC1 tumor-bearing animals randomized to receive sham surgery and αCTLA-4 (green lines, *n* = 6), αCTLA-4 alone (blue lines, *n* = 5) or control (black lines, *n* = 3). **f** Top: Experimental schema; bottom left: representative photographs of MOC1 tumor-bearing animals treated with combination α1A8 + αCTLA-4 with or without ND at day 15; bottom right: Tumor growth kinetics comparing combination treatment (green lines) and treatment after neck dissection (blue lines) (*n* = 5 animals/group). **g** Top left: Experimental schema for **h**; top right: representative photograph of a 4MOSC1 buccal tumor, day 16 (representative of *n* = 5 animals); bottom: representative gross specimen and immunohistochemical images of buccal tumors stained with H&E or anti-pan-CK antibody (representative of *n* = 5 tumors). **h** Top: Tumor growth kinetics from 4MOSC1 buccal tumor-bearing animals treated with αCTLA-4 (blue lines, *n* = 5) versus control (black lines, *n* = 5) or following ipsilateral (red lines, *n* = 5) versus contralateral ND (green lines, *n* = 5); bottom: representative images of 4MOSC1-LucOS tumor-bearing animals after indicated IO treatment, day 4 or 13 after tumor transplantation and surgery at day 3 (*n* = 4–5 animals/group). The differences between experimental groups were analyzed using simple linear regression analysis (**b**, **c**, left, **d**, **e**, **f**, **h**); and, survival analysis was performed using the Kaplan–Meier method and log-rank tests (**b**, **c**, right). All data represent averages ± SEM, except where indicated. ****P < 0.0001. ns not statistically significant. Source data are provided as a Source Data file.

Given the propensity for regional metastatic disease in our model (44% N+, see above, Fig. 1e), we hypothesized that lymphadenectomy sequenced before ICI therapy would improve tumor control and overall survival. To address this hypothesis, we performed neck dissection to ablate draining lymphatic basins in tumor-bearing animals prior to therapy with ICI (Fig. 2a). Unexpectedly, we found that neck dissection in advance of ICI in 4MOSC1-tongue tumor-bearing animals abolished the response to both αCTLA-4 and αPD-1 therapy, leading to significantly worse overall survival (Fig. 2b, c). In fact, we observed that neck dissection alone significantly impaired overall survival, suggesting that intrinsic antitumor immunity is compromised by lymphatic ablation (Supplementary Fig. 2A). As our model features a particular sensitivity to monotherapeutic αCTLA-4^30,32^, we leveraged this therapeutic modality as a surrogate for ICI immunotherapy to study the mechanistic underpinnings of how tumor-draining lymphatics mediate the antitumor response. Similar to ablation with neck dissection, high-dose large field single fraction radiation therapy, encompassing regional draining lymphatics (Supplementary Fig. 2B), in 4MOSC1-tongue tumor-bearing animals blocked the tumor response to αCTLA-4 ICI (Fig. 2d). To control for any confounding effects of surgery on the tumor response to therapy, we performed either sham mock lymphatic ablations to the neck or subtotal primary tumor resections followed by treatment with ICI. Neither sham neck surgery nor subtotal primary tumor surgeries influenced the response to ICI in vivo (Fig. 2e and Supplementary Fig. 2D). To demonstrate generalizability across syngeneic, orthotopic models of murine HNSCC, we employed the MOC1 preclinical HNSCC model that exhibits a near-complete response to the combination of αCTLA-4 and neutrophil-depleting antibody α1A8, which depletes myeloid-derived suppressor cells in the tumor immune microenvironment (TIME)^33^. Similar to 4MOSC1, neck dissection in animals with MOC1 tongue tumors failed to respond to combination α1A8 and αCTLA-4 immunotherapy (Fig. 2f).

Whether or not neck dissection targeting only tdLNs will compromise the response to ICI has not been reported. To address this, we developed a lateralized oral cavity tumor model in which 4MOSC1 is injected orthotopically into the buccal mucosa (Fig. 2g and Supplementary Fig. 2C), a space from which lymphatics drain exclusively to ipsilateral, regional cervical basins (see above, Fig. 1a, c). We observed that αCTLA-4 therapy leads to complete response in animals bearing 4MOSC1 buccal tumors and that this response is blocked with ipsilateral, but not contralateral, neck dissection (Fig. 2h and Supplementary Fig. 2E), supporting the critical role for tdLNs in mediating the response to ICI.

### Regional tumor-draining lymphatics coordinate antigen-specific CD8-driven immunity in the TIME

**Fig. 3:**
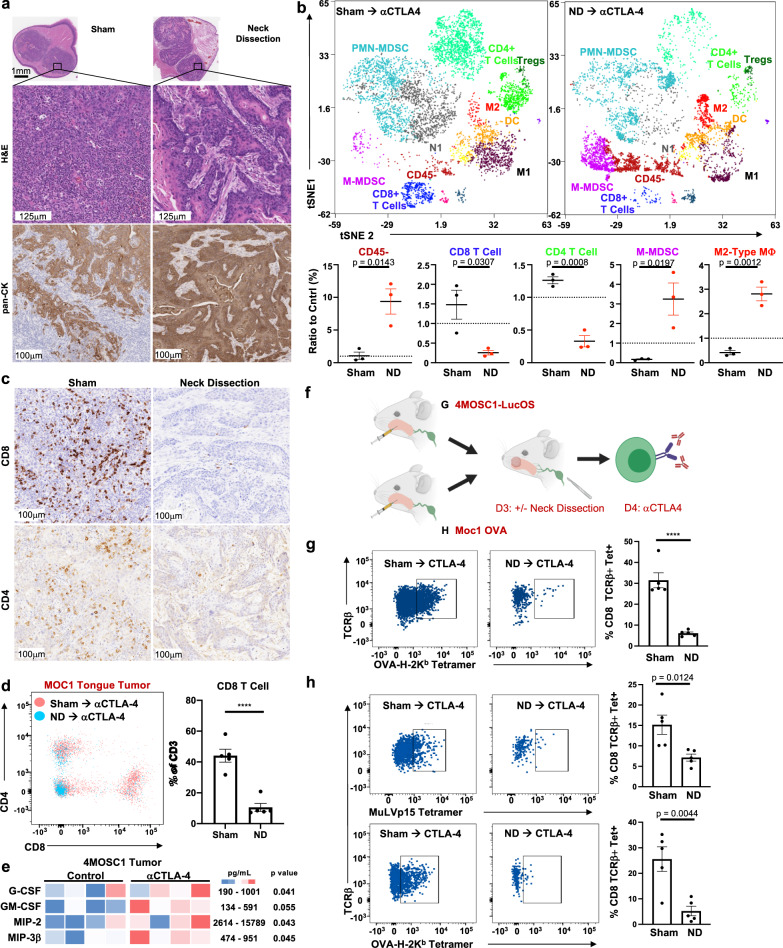
Regional tumor-draining lymphatics coordinate antigen-specific CD8-driven immunity in the tumor microenvironment. **a** Representative immunohistochemical images of 4MOSC1-tongue tumors from animals subjected to neck dissection or sham surgery followed by αCTLA-4 therapy, harvested at day 10. Shown are whole tumor sections, representative high-power H&E, and anti-pan-CK stained sections (representative of *n* = 5 tumors/treatment group). **b** Top: Representative tSNE plots shown from time-of-flight mass cytometry (CyTOF), comparing 4MOSC1-tongue tumors from animals subjected to neck dissection or sham surgery followed by αCTLA-4, harvested at day 10; bottom: quantification of selected populations identified in the TIME of the aforementioned groups (*n* = 3 samples/group). **c** Representative high-power IHC images probing for CD8+ or CD4+ cells in 4MOSC1-tongue tumors from animals subjected to neck dissection or sham surgery followed by αCTLA-4 therapy, harvested at day 10 (representative of *n* = 5 tumors/treatment group). **d** Flow plot and quantification comparing the CD4+ and CD8+ T-cell populations of MOC1 tongue tumors from animals subjected to neck dissection or sham surgery followed by αCTLA-4 therapy (red = sham surgery cohort, blue = neck dissection cohort, *n* = 5/group). **e** Heatmap comparing the expression of select chemokines and cytokines from the TIME of either control or αCTLA-4 treated 4MOSC1-tongue tumor-bearing animals at day 8 (*n* = 4/group). **f** Experimental schema—(**g**) 4MOSC1-LucOS or (**h**) MOC1-OVA tumors. Animals were randomized to receive sham surgery or neck dissection followed by treatment with αCTLA-4, after which tumors were harvested for flow cytometry to detect tumor-specific antigen tumor-infiltrating T cells. **g** Left: Representative flow cytometry plots and; right: quantification identifying TCRβ + OVA-H-2kb Tetramer+ CD8+ T cells from 4MOSC1-LucOS tumor-bearing animals harvested at day 10 after sham surgery or neck dissection and αCTLA-4 (*n* = 5/group). **h** Left: Representative flow cytometry plots; and right: quantification identifying TCRβ + MuLVp15 Tetramer+ or OVA-H-2kb Tetramer+ CD8+ T cells from MOC1-OVA tongue tumor-bearing animals harvested at day 10 after sham surgery or neck dissection and αCTLA-4 (*n* = 5/group). The differences between experimental groups were analyzed using independent, two-sided Student *t* tests (**b**, **d**, **e**, **g**, **h**). All data represent averages ± SEM, except where indicated. ****P < 0.0001. ns not statistically significant. Source data are provided as a Source Data file.

The extent to which and mechanisms by which tumor-draining lymphatics influence the tumor immune microenvironment during the response to ICI is not fully understood, although mounting evidence implicates their importance in antitumor immunity^34–38^. To address this, we profiled the TIME in 4MOSC1-tongue tumor-bearing animals treated with ICI after sham surgery or neck dissection. Histological analysis of the TIME with H&E and pan-CK staining reveals a predominantly lymphocytic infiltrate and less infiltrative cancer pattern in the primary tongue tumors of sham-operated animals compared to ND animals (Fig. 3a and Supplementary Fig. 3A). To define the changes in the TIME after neck dissection with greater resolution, we performed cytometry by time of flight (CyTOF), which revealed a tenfold over-representation of CD45- cells and a concomitant decrease in CD8 and CD4 T cells in ND versus sham cohorts, which was confirmed by IHC (Fig. 3b, c and Supplementary Fig. 3B). In addition, immunosuppressive myeloid populations—M-MDSCs and M2-Type macrophages—are overrepresented in the ND cohort (Fig. 3b). Of note, tumors for these experiments were harvested at a timepoint before which an objective change in tumor volume could be appreciated to control for any confounders attributable to tumors undergoing conspicuous, immune-mediated rejection (Supplementary Fig. 3C). The use of the MOC1 tongue tumor model revealed a similar reduction in CD8 T cells in the TIME following ND and treatment with combination αCTLA-4 and α1A8 (Fig. 3d).

To identify changes in the TIME secretome that precede changes in the intratumoral immune infiltration, we performed a cytokine/chemokine multiplex array comparing control and αCTLA-4-treated 4MOSC1 tumor-bearing animals (Fig. 3e). We found that the pro-inflammatory and myeloid-attractant cytokines, such as G-CSF and MIP-2 (CXCL2), and the DC-activating cytokines, GM-CSF^39^ and MIP-3β (CCL19)^40^, were significantly upregulated in tumors from ICI-treated animals compared to control.

Tumor-specific-antigen CD8 T (TSA-T) cells are appreciated as the primary drivers of antitumor immunity and responders to ICI therapy, as they can recognize and kill cancer cells expressing unique neoantigens^41^. To examine the infiltration of TSA-T cells into the TIME of animals receiving ICI therapy preceded by sham surgery or ND, we drove the expression of the model neoantigen, Ovalbumin (OVA), in our parent 4MOSC1 cell line to generate the 4MOSC1 pLenti-GFP-LucOS model (4MOSC1-LucOS; Supplementary Fig. 3D–F). 4MOSC1-LucOS and Moc1-OVA tongue tumor-bearing animals were treated with αCTLA-4 and subjected to sham surgery or ND (Fig. 3f). We found a roughly fivefold reduction in OVA-H-2K^b^ Tetramer+ CD8 T cells (OVA-Tet+ CD8 T cells) infiltrating into primary tongue tumors of ND-operated animals compared to sham-operated animals (Fig. 3g and Supplementary Fig. 3G, H). Further, we found that 4MOSC1-LucOS tumor-bearing animals having experienced a complete response to αCTLA-4 ICI therapy harbor OVA-Tet+ CD8 T cells that respond robustly to rechallenge with SIINFEKL-loaded bone marrow-derived dendritic cells, as assessed by IFNγ ELISpot assays (Supplementary Fig 3I, J). We validated these findings in MOC1-OVA tongue tumor-bearing animals, probing for TSA-CD8 T cells specific to either OVA or to the MuLVp15 antigen, which is expressed constitutively in this model^42^ (Fig. 3h and Supplementary Fig. 3H).

### Tumor-draining lymphatics harbor a population of conventional type I dendritic cells critical for the response to ICI

**Fig. 4:**
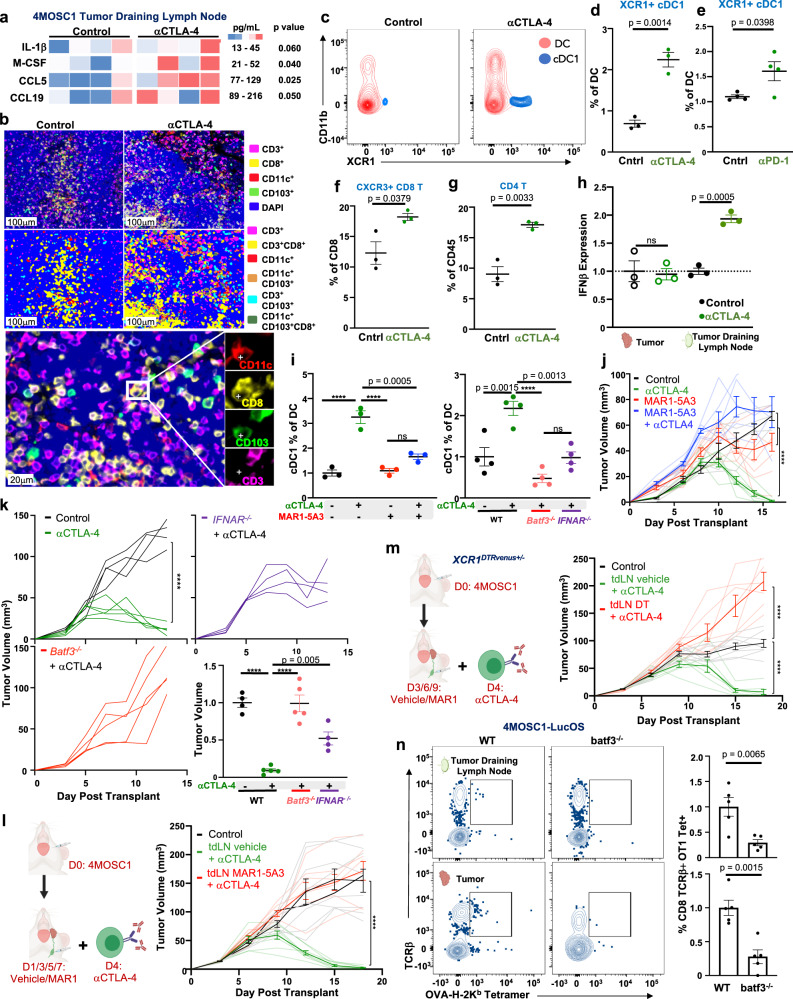
Tumor-draining lymphatics harbor a population of conventional type I dendritic cells critical for the response to ICI. **a** Heatmap comparing the expression of select chemokines and cytokines from tumor-draining lymph nodes of either control or αCTLA-4 treated 4MOSC1 tumor-bearing animals, day 8 (*n* = 4/group). **b** Representative multiplex immunofluorescence images identifying putative conventional type I dendritic cells (cDC1s) within the control or αCTLA-4 treated tdLNs from 4MOSC1 tumor-bearing animals, day 10 (representative of *n* = 10 tdLN/treatment group). **c** Representative flow cytometry contour plots identifying cDC1s (Ly6c-CD64-CD19-NK-CD11c + MHCIIhi CD11b-XCR1 +) from the tdLNs of control or αCTLA-4 treated 4MOSC1 tumor-bearing animals, day 10. **d** Quantification of cDC1s in tdLN after αCTLA-4 (*n* = 3). **e** Quantification of cDC1 in tdLN after αPD-1 (*n* = 4). **f** Quantification of activated CXCR3 + CD8+ T cells in the tdLNs of control or αCTLA-4-treated 4MOSC1 tumor-bearing animals, day 10 (*n* = 3). **g** Quantification of CD4+ T cells in the tdLNs of control or αCTLA-4-treated 4MOSC1 tumor-bearing animals, day 10 (*n* = 3). **h** IFNβ ELISA from tumor and tdLN from control or αCTLA-4 treated 4MOSC1 tumor-bearing animals, normalized to control, day 10 (*n* = 3). **i** Left: Quantification of cDC1s in the tdLNs of 4MOSC1 tumor-bearing WT animals treated with αCTLA-4 (green), MAR1-5A3 blocking antibody (red lines) or combination (blue lines) (*n* = 3 animals/group), day 10; (Right) and, in the tdLNs of WT (black), *batf3*^–/–^ (red), or *ifnar*^–/–^ (purple) animals treated with αCTLA-4 (*n* = 4 animals/group), day 10. **j** Tumor growth kinetics from 4MOSC1 tumor-bearing animals treated with αCTLA-4 (green lines, *n* = 7), MAR1-5A3 blocking antibody (red lines, *n* = 6), combination therapy (blue lines, *n* = 6) or control (black lines, *n* = 6). **k** Tumor growth kinetics from 4MOSC1 tumor-bearing WT control (black lines, *n* = 4) and αCTLA-4 treated animals (green lines, *n* = 5) versus αCTLA-4 treated *batf3*^–/–^ (red lines, *n* = 5) or *ifnar*^−/−^ (purple lines, *n* = 4); bottom right: tumor volume normalized to control at day 13. **l** Left: Experimental schema: 5 μg of MAR1-5A3 blocking antibody or vehicle was injected into the tdLN every 2 days, beginning day 1, for a total of 4 doses. Following the development of conspicuous tumors, animals were randomized to receive αCTLA-4. Right: Tumor growth kinetics from 4MOSC1 buccal tumor-bearing animals treated with αCTLA-4 ICI and tdLN-injected local IFNAR blockade (red lines) or vehicle (green lines) (*n* = 8 animals/treatment group). **m** Left: Experimental schema: 1 μg of diphtheria toxin or vehicle was injected into the tdLN every 3 days, beginning on day 3, for a total of three doses. Following the development of conspicuous tumors, *XCR1*^*DTRVenus+/–*^ animals were randomized to receive αCTLA-4. Right: Tumor growth kinetics from 4MOSC1 buccal tumor-bearing animals treated with αCTLA-4 ICI and tdLN-injected local diphtheria toxin (red lines, *n* = 7) or vehicle (green lines, *n* = 7) versus control (black lines, *n* = 8). **n** Left: Representative flow cytometry plots and; right: quantification identifying TCRβ + OVA-H-2kb Tetramer+ CD8+ T cells from 4MOSC1-LucOS tongue tumor-bearing WT or *batf3*^−/−^ animals, day 10 (*n* = 5). The differences between experimental groups were analyzed using independent, two-sided Student *t* tests (**a**, **d**–**h**, **n**, right), one-way ANOVA (**i**, **k**, bottom right) or simple linear regression analysis (**j**–**m**). All data represent averages ± SEM, except where indicated. ****P < 0.0001. ns not statistically significant. Source data are provided as a Source Data file.

We next turned our attention to the tumor-draining lymph nodes that are targeted with neck dissection. An analysis of the secretome in the tdLN after initiation of αCTLA-4 therapy revealed an upregulation of signals to recruit dendritic cells—G-CSF and CXCL2—and others which activate, mature, and potentiate dendritic cells—M-CSF and IL-1β^40,43–45^ (Fig. 4a). In particular, M-CSF has a previously described role as a conventional dendritic cell poietin^46^; and, IL-1β is known to be a critical factor to bridge innate and adaptive antipathogen immunity by driving DC maturation and IL-12 secretion, which in turn facilitate T-cell priming^47,48^. Given the requirement of the tdLN for TSA-T-cell infiltration into tumors and the upregulation of DC-potentiating chemokines and cytokines after ICI, we speculated that DCs within tumor-draining lymphatics are responsible for mounting antitumor adaptive immunity and mediating the host response to ICI.

To explore this, we examined cervical, draining lymphatic basins during the response of 4MOSC1-tongue tumors to ICI. We focused on conventional type I dendritic cells (cDC1s), which are recognized as the most potent cross-presenting immune effectors with documented roles in priming antigen-specific T-cell response during antipathogen and antitumor responses^49–51^. Multiplex immunofluoresence analysis of tumor-draining lymph nodes (tdLNs) in 4MOSC1-tongue tumor-bearing animals reveals an accumulation of cDC1s (CD11c^+^CD8^+^CD103^+^CD3^−^) in αCTLA-4 treated animals compared to control-treated animals (Fig. 4b). To quantify the relative abundance of cDC1s in tdLNs of animals treated with ICI, we harvested tdLNs and quantified major DC populations by flow cytometry (Fig. 4c and Supplementary Fig. 4A, B). By design, tdLNs were harvested at a time point prior to observable changes in tumor growth kinetics between control and treatment groups (Supplementary Fig 4C). We found that cDC1s, defined as CD45^+^CD64^−^Ly6C^−^CD11c^+^MHCII^hi^CD11b^−^XCR1^+^ cells^52,53^, are increased twofold following therapy with ICI (Fig. 4d, e). Concomitantly, the activated CD8 T cell (CXCR3+) and total CD4 T-cell populations were significantly overrepresented in ICI-treated animals compared to control within the tdLN (Fig. 4f, g).

Type I interferon (IFN-I) signaling is central to host antitumor immunity, principally through the licensing of cDC1 cells^51,54–56^. To examine the role of IFN-I programs in tdLNs in tumor-bearing animals during the response to ICI, we measured the expression of IFNβ by ELISA in tdLN. We found that IFNβ expression is doubled in the tdLNs, but not the tumors, of ICI-treated animals, suggesting that IFNβ may serve to license LN-associated cDC1s, which, in turn, enhance TSA-CD8 T-cell priming during ICI treatment (Fig. 4h). To explore this possibility, we measured the abundance of cDC1 in tdLNs following IFNAR blockade—achieved with either pharmacological inhibition or in *ifnar*^−/−^ genetically engineered mouse models (GEMMs)—and found that tdLN-associated cDC1s are significantly reduced in both cases (Fig. 4i, right and left, respectively). To more precisely identify the role that IFNAR signaling has in cDC1-mediated T-cell priming, we employed the bone marrow-derived, induced cDC1 (iDC) in vitro model^57,58^. This in vitro model generates bonafide *batf3* cDC1 cells capable of robust cross-presentation following induction with GM-CSF and Flt3L in vitro (Supplementary Fig. 4D–F). To examine the role of IFNAR signaling in licensing iDCs to prime and activate TSA-CD8 T cells, we measured the expression of double positive IL-2+ IFNγ + OT-1 T cells, which feature clonotypically restricted TCRs against the model antigen Ovalbumin, co-cultured with activated iDCs cross-presenting SIINFEKL peptide. We found that OT-1 T cells cultured with SIINFEKL + iDCs readily activate to express IL-2 and IFNγ (Supplementary Fig. 4G). However, the addition of MAR1-5A3 to block IFNAR signaling significantly restrains TSA-CD8 T-cell activation (Supplementary Fig. 4G). Indeed, we show that IFN-I blockade significantly reduces the capacity of OT-1 T cells to kill OVA-expressing target cells following co-culture with activated SIINFEKL cross-presenting iDCs (Supplementary Fig. 4H).

We next hypothesized that IFNAR signaling and cDC1s are central to the tumor response to ICI therapy in our model. To explore this hypothesis, we transplanted 4MOSC1 into the tongues of WT, *Batf3*^−/−^—which lack cDC1s^50^—or *IFNAR*^−/−^ animals and treated with αCTLA-4. We found blockade of IFNAR signaling—achieved with either pharmacological blockade or in *ifnar*^−/−^ GEMM animals—in 4MOSC1-tongue tumor-bearing animals fail to respond to αCTLA-4, suggesting that IFNAR signaling is necessary for the host response to αCTLA-4 (Fig. 4j, k and Supplementary Fig. 4I). Similarly, batf3-deficient animals lacking cDC1s fail to respond to αCTLA-4 (Fig. 4k).

Next, we sought to determine whether the requirement for IFNAR signaling and cDC1s in the host response is exclusive to the tdLN. To accomplish this, we developed a model to locally deliver minute payload via microsyringe injections directly into the tumor-draining lymph nodes of 4MOSC1 buccal tumor-bearing animals (Supplementary Fig. 4J). Importantly, we found that serial microsyringe injection of vehicle into the tdLN did not compromise the overall anatomy or integrity of the tdLN nor the primary tumor (Supplementary Fig. 4K). We first employed this model to determine the necessity of tdLN-exclusive IFNAR signaling for the host response to ICI therapy. Through repeated injections of MAR1-5A3 into the tdLN of 4MOSC1 buccal tumor-bearing animals, we observed the successful blockade of IFNAR exclusively in the tdLN but not the tumor (Supplementary Fig. 4L, M). tdLN IFNAR blockade compromised the primary tumor response to αCTLA-4 ICI (Fig. 4l). Similarly, to determine the necessity of cDC1s in the tdLN for the host ICI response, we employed the *XCR1*^*DTRVenus*^ GEMM to achieve cDC1-specific depletion by exposure to diphtheria toxin (DT)^59^. Delivery of DT into the tdLNs of 4MOSC1 buccal tumor-bearing *XCR1*^*DTRVenus+/–*^ mice led to a localized depletion of cDC1s and the loss of the host ICI response (Fig. 4m and Supplementary Fig. 4N). As a control, local tdLN injection with vehicle did not compromise the host response to ICI therapy (Fig. 4l, m, green lines).

Next, we assessed whether cDC1s can influence the generation and subsequent intratumoral infiltration of TSA-T cells, even in absence of ICI therapy. We found that 4MOSC-LucOS tumor-bearing *batf3*-deficient animals harbor fewer TSA-T cells in both the tdLN and tumor microenvironments (Fig. 4n).

### Rational IO treatment sequencing drives primary tumor treatment responses and immunosurveillance to protect against locoregional nodal metastasis

**Fig. 5:**
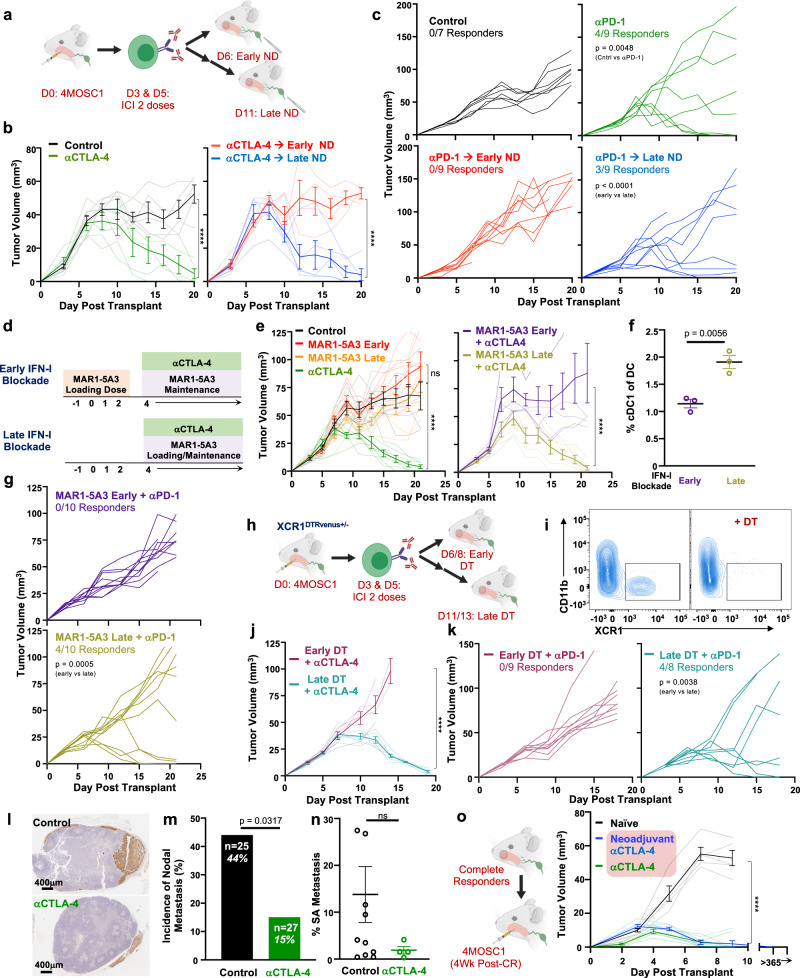
Rational IO treatment sequencing drives primary tumor treatment responses and immunosurveillance to protect against locoregional nodal metastasis. **a** Experimental schema for **b**, **c**. **b** Tumor growth kinetics from 4MOSC1 tumor-bearing control animals (black lines, *n* = 5) or αCTLA-4-treated animals (green lines, *n* = 5), followed by either early (red lines, *n* = 4) or late (blue lines, *n* = 5) neck dissection. **c** Tumor growth kinetics from 4MOSC1 tumor-bearing control animals (black lines, *n* = 7) or αPD-1-treated animals (green lines, *n* = 9), followed by either early (red lines, *n* = 9) or late (blue lines, *n* = 9) neck dissection. **d** Experimental schema for **e**, **g**. **e** Left: Tumor growth kinetics from 4MOSC1 tumor-bearing control animals (black lines, *n* = 7) or αCTLA-4 treated animals (green lines, *n* = 7), early IFNAR blockade animals (red lines, *n* = 7), or late IFNAR blockade animals (orange lines, *n* = 7). Right: Tumor growth kinetics from 4MOSC1-tongue tumor-bearing animals treated with αCTLA-4 after receiving early IFNAR blockade (purple lines, *n* = 6) or late IFNAR blockade (dark yellow lines, *n* = 7). **f** Percent cDC1s in the tdLNs of 4MOSC1 tumor-bearing animals treated with αCTLA-4 after receiving early (purple lines) or late (dark yellow lines) IFNAR blockade (*n* = 3/group). **g** Tumor growth kinetics from 4MOSC1 tumor-bearing animals treated with αPD-1 after receiving early (purple lines) or late (dark yellow lines) IFNAR blockade (*n* = 10/group). **h** Experimental schema for **j**, **k**. **i** Representative flow cytometry plots demonstrating the conditional depletion of cDC1s in αCTLA-4 treated 4MOSC1-tongue tumor-bearing *XCR1*^*DTRvenus+/−*^ animals one day after systemic delivery of diphtheria toxin (DT) (representative of *n* = 5 independent samples/group). **j** Tumor growth kinetics from 4MOSC1 umor-bearing *XCR1*^*DTRvenus+/−*^ animals treated with αCTLA-4 and (purple lines) or late (turquoise lines) DT (*n* = 3 animals/treatment group). **k** Tumor growth kinetics from 4MOSC1 tumor-bearing *XCR1*^*DTRvenus+/−*^ animals treated with αPD-1 and early (purple lines, *n* = 9) or late (turquoise lines, *n* = 8) DT. **l** Representative immunohistochemical images of tdLNs stained with pan-CK rom 4MOSC1-tongue tumor-bearing animals treated with two doses of αCTLA-4 compared to control, day 11, with; (**m**) Incidence of metastatic disease in tdLNs (*n* = 25–27 individual tumor-draining lymph nodes/group); and, (**n**) Quantification of the burden of metastatic disease among tdLNs with occult nodal disease from 4MOSC1 tumor-bearing animals treated with two doses of αCTLA-4 (*n* = 4) compared to control (*n* = 11). **o** Left: Experimental schema; right: tumor growth kinetics in naive (black lines) or previous complete responders after either six doses (green lines) or two doses of αCTLA-4 (blue lines) (naive *n* = 5/group; long-term survival, *n* = 3/group). The differences between experimental groups were analyzed using independent, two-sided Student *t* tests (**f**, **n**), fisher’s exact test (**m**) or simple linear regression analysis (**b**, **c**, **e**, **g**, **j**, **k**, **o**). All data represent averages ± SEM, except where indicated. ****P < 0.0001. ns not statistically significant. Source data are provided as a Source Data file.

Given our observations that tdLNs are required for the response to ICI, we hypothesized that delivering ICI in advance of lymphatic ablation will achieve an optimal therapeutic response. To identify effective sequences of IO treatment, we developed a neoadjuvant model in which animals with established tongue tumors are treated with two doses of ICI prior to neck dissection, which is performed either 1 or 6 days after the final dose of ICI—day 6 or day 11 post tumor implantation, respectively (Fig. 5a). The delayed neck dissection timepoint was selected intentionally to precede detectable changes in tumor volume between control and treatment groups but to follow the time at which we observe TSA-T cell infiltration into tumors. We found that two doses of αCTLA-4 is sufficient to mediate a complete response, which is abolished by early but not late ND (Fig. 5b and Supplementary Fig. 5A). Similarly, early but not late neck dissection blocks the tumor response to αPD-1 ICI (Fig. 5c). This empiric model of treatment sequencing enabled us to explore the timing of the contribution of IFNAR signaling and cDC1s to the host response to therapy. To address this, we blocked IFNAR signaling pharmacologically with systemic delivery of the depleting antibody, MAR1-5A3, either coincident with tumor implantation or delayed to co-occur with ICI delivery (Fig. 5d). We found that delaying IFNAR blockade permits the tumor response to therapy and leads to a twofold increase in tdLN-associated cDC1s (Fig. 5e–g and Supplementary Fig. 5B). In a similar fashion, to determine the temporal role for cDC1s during the ICI response, we employed the *XCR1*^*DTRVenus*^ GEMM to deplete cDC1 with diphtheria toxin either 1 or 6 days after the final dose of ICI (Fig. 5h, i)^59^. We found that while early cDC1 depletion prevents the tumor response to ICI, the tumor response to treatment is preserved with delayed cDC1 depletion (Fig. 5j, k).

Locoregional (LR) recurrence for HNSCC patients remains an outstanding clinical problem, with significant recurrence rates in patients with advanced disease^27,60^. Our 4MOSC1 orthotopic model features a regional metastatic burden (~44% in tdLNs, Fig. 1f), which is similar to the incidence of regional metastasis observed in oral SCC patients^61^. While recent reports from emerging neoadjuvant “window of opportunity” immunotherapy trials in HNSCC are reporting improvements in primary tumor control^62^, rates of control for LR disease with upfront ICI remain unknown. To explore this, we probed, first, for the presence of metastasis in tdLNs; and, second, for the overall burden of disease in tdLNs with occult metastasis (met+), as assessed by IHC with pan-CK in tdLNs harvested after surgery at day 10—a timepoint at which the primary tumor burden between treatment groups is not significantly different (Fig. 5l–n). We observed that αCTLA-4-treated tongue tumor-bearing animals harbor a nearly three-fold reduction in overall metastatic burden compared to control (15% [*n* = 27] vs 44% [*n* = 25] regional met+, *P* = 0.0317). In addition, analysis of disease burden among met+ tdLNs reveals that αCTLA-4 reduces the burden of disease as assessed by the percent of surface area with the disease per individual met+ LN. This provides evidence that ICI may control both the development and progression of regional metastasis in HNSCC.

In addition, we sought to address whether a complete primary tumor response to neoadjuvant immunotherapy might also confer durable immunity in HNSCC. To test this, we employed a model in which 4MOSC1-tongue tumor-bearing animals are treated with αCTLA-4, which leads to complete rejection in immunocompetent hosts. Re-challenging these complete responder animals with parental 4MOSC1 leads to rapid and complete tumor clearance, similar to traditional tumor vaccination models with implantation of irradiated tumor cells^30^. We observed that animals with complete response to neoadjuvant therapy are imbued with long-lasting antitumor immunity and reject rechallenge with parental tumors (Fig. 5o). In tandem, we sought to determine whether durable anticancer immunity can be conferred distal to and independent of regional, cervical lymphatics. To address the necessity of tumor-draining lymphatics after a complete response to IO therapy, we performed neck dissections in our ICI-treated animals that had achieved complete responses. We found that ablation of regional tumor lymphatics in complete responder animals does not impair host immunity, as they robustly reject tumor rechallenges (Supplementary Fig. 5C). Together, these finding suggests that upfront IO therapy, delivered prior to lymphatic ablation, may confer immunologic control of locoregional recurrences by driving immunosurveillance programs from the periphery.

### Lymphatic-sparing IO therapy mobilizes peripheral antitumor immunity

**Fig. 6:**
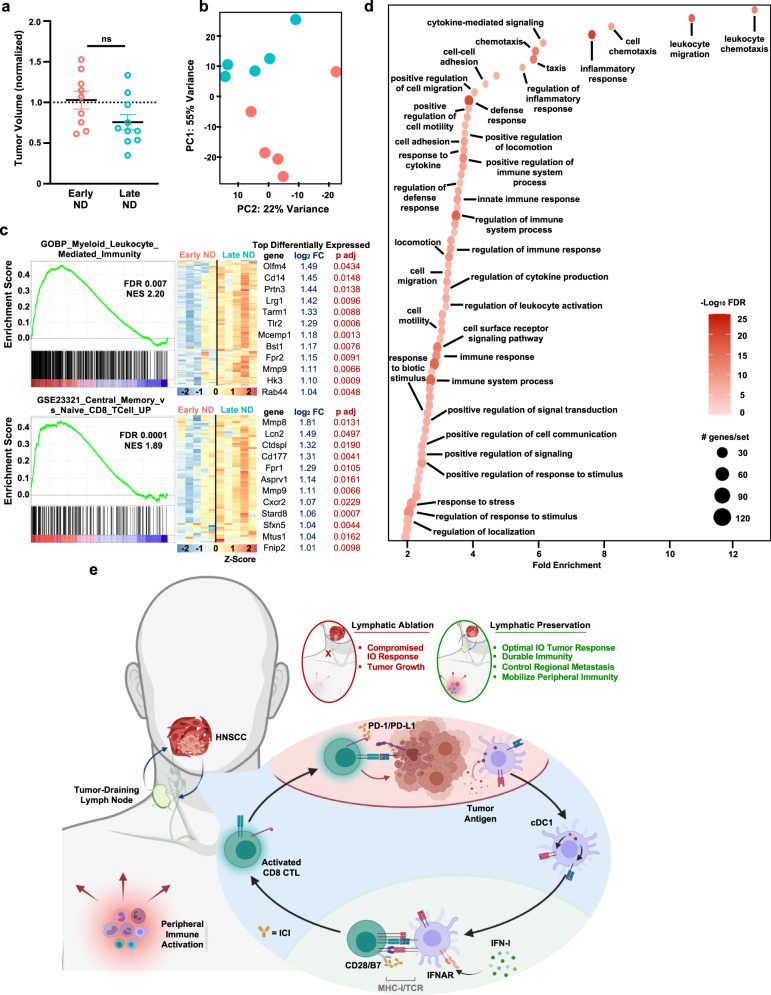
Lymphatic-sparing IO therapy mobilizes peripheral antitumor immunity. **a** Normalized tumor volumes on day 9 or 10 after orthotopic transplantation of 10^6^ 4MOSC1 tumor cells and treatment in vivo with two doses of αCTLA-4 followed by either an early (red, *n* = 9) or late (blue, *n* = 10) neck dissection—day 6 versus day 11. **b** Principal component analysis plot from whole blood RNA sequencing, performed at day 10 after transplantation of 10^6^ 4MOSC1 tumor cells and treatment in vivo with two doses of αCTLA-4 followed by either an early (red) or late (blue) neck dissection—day 6 versus day 11, calculated and plotted with DESeq2, *n* = 5. **c** Left: Representative gene set enrichment mountain plots of differentially expressed genes; and right: corresponding heatmaps depicting the row normalized Z-score of the top differentially expressed genes from those gene sets identified in analysis of whole blood RNA sequencing after transplantation of 10^6^ 4MOSC1 tumor cells and treatment in vivo with two doses of αCTLA-4, followed by either an early (red), or late (blue) neck dissection. **d** Bubble plot illustrating the top hits from a Gene Ontology analysis, illustrating GO hits enriched in late vs early ND treatment sequencing groups as described in Fig. 5a (log_2_FC > 1, adjusted *P* value < 0.05 upregulated genes identified in analysis of whole blood RNA sequencing; *n* = 5). **e** Cartoon describing the central role that the tumor-draining lymph node plays in the response to ICI therapy and the outcome of rational, lymphatic-preserving treatment sequencing in HNSCC; see text for details. The differences between experimental groups were analyzed using independent, two-sided *t* tests (**a**), DESeq2 (**c**) or Log_2_FC *P* < 0.05 (**d**). All data represent averages ± SEM, except where indicated. ns   not statistically significant. Source data are provided as a Source Data file.

In order to explore the host peripheral immune response to IO treatment sequencing, we analyzed the transcriptome of whole blood from animals receiving ICI and either early or late neck dissection. We hypothesized that sequencing neck dissection in a delayed fashion after ICI, which leads to complete primary tumor response and regional control of metastasis, would concomitantly lead to peripheral immune responses that are not observed with immediately sequenced neck dissection. For these studies, we collected whole blood for RNAseq at a timepoint following delayed neck dissection but preceding a significant change in the tumor volume between treatment cohorts (Fig. 6a). Principle component analysis of the transcriptome from whole blood between these groups reveals that the timing of surgery alone alters the global transcriptome of the peripheral immune compartment (Fig. 6b). To identify patterns of transcriptomic changes that can account for the divergence between groups, we performed gene set enrichment analysis (GSEA)^63,64^, employing the Gene Ontology (Biological processes) and ImmuneSigDB collections^65^, which identified gene sets related to host immunity as those which are most significantly different. Illustratively, we show GSEA analysis featuring signatures related to myeloid activation and T-cell immunity in late neck dissection treatment sequences (Fig. 6c). To explore this further, we employed gene ontology (GO) analysis, focusing on significantly upregulated, differentially expressed genes (*P*_adj_ < 0.05, Log_2_FC > 1, minimum gene/set 30). GO analysis indicated that treatment sequences in which lymphatic ablation is delayed relative to ICI engenders a robust host immune response, during which immune effectors are activated, and programs of defense are marshaled (Fig. 6d). These findings suggest that the successful host response to ICI extends from the locoregional space to the periphery (Fig. 6e).

## Discussion

Collective clinical experience with IO therapy, while promising, has demonstrated limitations in both r/m and locally advanced HNSCC. In the r/m setting, αPD-1 ICI yields only limited responses^18–20^, and αCTLA-4 ICI, which has demonstrated benefit for other solid tumors with similar immune infiltrate^22^ and mutational burden^21^, has failed to demonstrate clinical benefit in HNSCC^23,66^. More recently, in the curative-intent setting for locally advanced disease, emerging evidence from phase III clinical trials now indicates that adding αPD-1 ICI to conventional therapies confers no additional benefit^25^. Together, these data raise the possibility that the current standard of care therapies for HNSCC may interfere with the host’s ability to respond to immune-oncology therapy. Specifically, our hypothesis is that locoregional therapies for HNSCC, which by design ablate locoregional lymphatics, compromise host immunity and the tumor response to ICI.

Primary murine preclinical models, particularly syngeneic tumor models, have afforded tremendous insight into the dynamics of tumor–immune interactions and the design of effective IO therapies^67–71^. Most recently, “next-generation” syngeneic preclinical models have been characterized, featuring mutational signatures analogous to that of their human cancer counterparts, transplantability into orthotopic sites, and profiles of immune infiltration and response to IO therapy similar to that observed clinically^5,30,72–74^. As such, these sophisticated preclinical systems are uniquely suited to address the contemporary challenges of deconstructing cancer-immune dynamics and designing rational new IO therapies. Leveraging the power of these preclinical models, many conceptual advances have been made in understanding how conventional anticancer therapies influence the efficacy of immunotherapy and in optimizing those interactions to yield improved oncologic outcomes^67,75^. And, there is mounting evidence suggesting that the efficacy of ICI may depend upon an intact tumor-lymphatic axis^34–38^. However, to date, a tractable and clinically relevant model ablating tumor-draining lymph nodes—and, thus reflecting standard of care clinical practice—to simultaneously interrogate local, regional and systemic immune responses and tumor–immune interactions during IO therapy in HNSCC has not been available. This has precluded the ability to address the dynamic host response to multimodal therapy. Specifically, translatable models in which standard of care lymphablative therapies can be employed are lacking. To address this and begin to answer the outstanding questions regarding the design of optimal IO therapy, we developed a preclinical system in which several key elements of modern IO therapy for HNSCC have been applied to a next-generation, syngeneic, translational model. Here, we employ a clinically relevant, syngeneic tobacco-signature 4MOSC oral SCC preclinical model to address our hypothesis and, ultimately, to define rational IO treatment sequences for locoregional HNSCC. We mapped tumor-draining lymphatics and developed models for regional nodal ablation with surgery or ablative radiation therapy. Our tumor model faithfully drains to defined regional, nodal basins and bears a propensity for occult regional metastasis similar to that appreciated clinically^27,29^. Remarkably, we found that ablating tumor-draining lymphatics eradicates the tumor response to ICI and leads to significantly worse overall survival. Within the TIME, lymphatic ablation reverses the beneficial antitumor effect of ICI; and, instead, promotes immunosuppression with reduction of tumor-specific antigen CD8 T-cell infiltration. Ultimately, we were able to define a rational IO therapeutic strategy that mobilizes peripheral immunity, achieves an optimal primary tumor response, confers durable immunity and controls the development of regional lymphatic metastasis.

Principal among the challenges impeding the rational design of maximally effective IO therapy is how to combine and sequence treatments with respect to the host multisystem tumor-lymphatic-vascular anticancer response^67^. Traditionally, advances in treating locoregional HNSCC have been facilitated by the stepwise addition of novel treatments onto the framework of existing standard of care treatments. However, the introduction of immunotherapies into standard of care management for HNSCC has not motivated practice-defining, parallel efforts to explore optimal treatment sequencing. Rather, the landmark clinical trials defining how we employ IO therapy in HNSCC have recruited patients who have contemporaneously or previously received lymphablative therapy, which we hypothesized would compromise the tumor response to IO therapy. Indeed, taking advantage of the high sensitivity of our preclinical model to αCTLA-4, which may be in part due to regulatory T-cell depletion not observed with the human targeting antibody^76^, the data presented here support that standard locoregional, lymphablative therapies for HNSCC compromise the primary tumor response to ICI, thereby providing insights that can be applied to define rational IO treatment sequences that optimize antitumor immunity orchestrated by regional lymphatics. Interestingly, these findings may provide a rationale for re-exploring the use of αCTLA-4 for HNSCC patients. Although αCTLA-4 delivered in tandem with standard therapies has failed to demonstrate benefit in HNSCC^24,66^, our findings suggest that αCTLA-4 may prove efficacious if sequenced in advance of ablative therapies. Additionally, we have extended our key observations in this work with αPD-1, which is approved for HNSCC, suggesting that rational treatment sequencing with delayed lymphatic ablation may also increase αPD-1 IO responses in HNSCC patients. Aligned with this prediction, a recently reported phase I clinical trial in patients with previously untreated locally advanced HNSCC delivered αPD-1 ICI in combination stereotactic radiation directed only to the gross tumor—hence, sparing uninvolved draining regional lymphatics—followed by surgical resection, resulted in increased complete pathological responses and clinical to pathological downstaging of most patients^77^. This recent report and our current preclinical work support further clinical evaluation of the role of neoadjuvant or induction ICI for newly-diagnosed HNSCC with the goal of improving overall treatment responses and regression-free survival. In more general terms and from a translational standpoint, our findings suggest that IO therapy may need to be sequenced with ICI delivered sufficiently in advance of standard oncologic therapies, which by design compromise tumor-draining lymphatics—a strategy that may extend well beyond HNSCC to improve outcomes for a host of other cancers patients. Such a treatment sequencing approach may enable optimal primary tumor responses to IO therapy and, simultaneously, initiate regional and peripheral programs of immunosurveillance, conferring durable anticancer immunity.

Mechanistically, our preclinical studies strongly support the rationale for upfront lymphatic-preserving IO therapy, particularly to harness the endogenous IFN-I- and cDC1-driven antitumor response in the immediate period following delivery of ICI. We found that αCTLA-4 ICI marshals a robust locoregional antitumor immune response. An analysis of the TIME and tdLN after αCTLA-4 ICI revealed a significant upregulation in patterns of cytokines and chemokines known to drive antitumor immunity and, in some cases, under current investigation as adjuncts to IO therapy both as single agents and in combinations for various cancers^43^. Moreover, the combination of cytokines and chemokines whose expression increased following αCTLA-4 ICI —GM-CSF/CCL19 within the TIME and G-CSF/CXCL2 and M-CSF/IL-1β within the tdLN—are known to potently drive DC recruitment, maturation, and function, specifically antigen processing and cross-presentation^40,43–45^. In particular, M-CSF has a previously described role as a conventional dendritic cell poietin^46^; and IL-1β is known to be a critical factor to bridge innate and adaptive antipathogen immunity by driving DC maturation and IL-12 secretion, which in turn facilitate T-cell priming^47,48^. Taken together, these findings suggest that DC mobilization and activation may be central to the tumor response to αCTLA-4. In support, we find that conventional type I dendritic cells and IFN-I signaling within tdLNs are critical for the priming and subsequent tumor-infiltration of tumor-specific antigen cytotoxic lymphocytes, which aligns more broadly with a host of reports defining the essential function of cDC1s and IFN-I signaling in host immunity^51,53,54,56,78,79^. While we find that IFNAR signaling and cDC1s—specifically within the tumor-draining lymph node—are critical for the host ICI response, the successful antitumor ICI response is likely multifactorial and will require additional investigation.

Recent reports from the clinical literature have demonstrated a positive correlation between the onset of immune-related adverse events (irAEs) and the tumor response to ICI^80–82^, suggesting that effective IO therapy should be reflected and manifest proximal to the TIME. In line with this observation, we found that lymphatic-sparing treatment sequencing activated both regional and peripheral host antitumor immunity. Unexpectedly, we observed a three-fold reduction in the incidence of occult regional lymphatic metastasis following therapy with αCTLA-4 therapy, an observation that occurred coincident in time with primary tumor responses in animals receiving lymphatic-sparing treatment sequences. Moreover, our analysis of the transcriptome of whole blood revealed a robust activation in programs of immunity, defense, and mobilization of immune effectors only in those animals receiving upfront lymphatic-preserving IO therapy. Lastly, we observed that animals with complete primary tumor responses after ICI and delayed lymphatic ablation are imbued with durable antitumor immunity. Collectively, these findings suggest that preserving lymphatics during IO therapy not only enhances primary tumor responses but also unleashes a regional and systemic host antitumor immunity.

The mechanisms by which immunotherapies exert anticancer activity fundamentally diverge from those of conventional oncologic therapies. While conventional therapies specifically target cancer cells to affect cytotoxicity or arrest growth, immunotherapies harness the host immune response to recognize, surveil and attack cancer cells. This complex immunologic process is dependent upon multiple organ systems— hematologic, lymphatic, and vascular, among others—working dynamically and in conjunction with one another. Our findings suggest that in the conception and implementation of future IO therapeutic strategies, it is imperative that conventional and IO therapies be rationally designed and sequenced to achieve locoregional and distant cancer control while intentionally preserving the host’s ability to mount an effective antitumor response.

## Methods

All the animal studies were approved by the University of California San Diego (UCSD) Institutional Animal Care and Use Committee (IACUC, protocol #S15195); all experiments adhere with all relevant ethical regulations for animal testing and research.

### Study design

ARRIVE 2.0 guidelines^83^ for reporting animal research were employed as follows:

#### Sample size

The sample size for each experiment was selected in accordance with historical data from the preclinical models employed in order to achieve significance; described in detail in each experiment and the statistical analysis section below.

#### Rules for stopping data collection

In the case of in vivo experiments, stopping rules were pre-approved according to the University of California San Diego (UCSD) Institutional Animal Care and Use Committee (IACUC), with protocol ASP #S15195 (described below).

#### Data inclusion/exclusion

All data collected was included and represented in the main figures or supplementary materials.

#### Outliers

Outliers were included in the reported data.

#### Replicates

All experiments, when feasible, were repeated at least twice and reproducibility confirmed; in all possible instances, data from repeat experiments is represented.

#### Research objectives

The research objective did not alter and is as follows: to provide a mechanistic understanding of how standard oncologic therapies targeting regional lymphatics impact the tumor response to immune-oncology therapy in order to define rational treatment sequences that mobilize systemic antitumor immunity, achieve optimal tumor responses, confer durable antitumor immunity, and control regional metastatic disease.

#### Research subjects

We employed translational preclinical models of HNSCC, as described below.

#### Experimental design

This work represents a controlled, laboratory investigation involving preclinical models of HNSCC. Treatments applied were designed to deliberately model current clinical therapies for HNSCC patients. In general, endpoints for studies presented include tumor growth kinetics, survival analyses and a spectrum of immunological analyses.

#### Randomization

All in vivo experiments were randomized by tumor volume prior to initiation of treatment or data collection.

### Reagents

Anti-PD-1 antibody (clone J43, BE0033-2), anti-CTLA-4 (clone 9H10, BP0131), anti-Ly6G (clone 1A8, BE0075-1), and IFNAR depleting antibodies (Clone MAR1-5A3, BE0241) were purchased from Bio X Cell (West Lebanon, NH). Fluorochrome-conjugated antibodies were purchased from BD Biosciences (San Jose, CA) and BioLegend (San Diego, CA). pHAGE PGK-GFP-IRES-LUC-W (#46793), Lenti-LucOS (#22777), and pLenti-CMV GFP-DEST (# 19732) were obtained from Addgene. All other chemicals and reagents were from Sigma-Aldrich (St. Louis, MO) unless indicated.

### Murine cervical lymphatic mapping

In all, 5% Evans Blue dye (Sigma, St. Louis, MO) in 40 μL phosphate-buffered saline or LYVE-ef660 (ThermoFisher Scientific, San Diego) diluted 1:50 into 40 μL phosphate-buffered saline was injected into the various head and neck subsites in the mouse—oral tongue, buccal mucosa, base of tongue, and retroauricular space—using a 1.0 mL syringe with a 31 g ½” needle (Becton-Dickinson, Franklin Lakes, NJ). Following induction with 3% isoflurane, the diluted dye was injected into the aforementioned head and neck subsites. In both cases, dye or LYVE antibody was visually confirmed to enter the specified tissues without backflow or leakage.

For mapping with Evans Blue dye, after 5–10 min following injection, the cervical space was explored as follows: (i) the anesthetized mouse was positioned and draped and prepped in a sterile fashion; (ii) under ×8 operative microscopy, the skin was incised sharply in the midline with straight microscissors; (ii) skin flaps were bluntly elevated laterally to broadly expose the cervical space spanning from the angle of the mandible bilaterally to the clavicles. Superficial lymphatic basins were encountered immediately deep to the dermis and adjacent the superolateral aspects of the submandibular glands. Reflecting the submandibular glands and superficial lymphatic basins laterally revealed the jugular venus plexus and deep lymphatic basins nested within the jugular vascular confluence and atop the floor of the neck. Dyed lymphatic vessels and basins draining from injected head and neck subsites were readily apparent during this dissection. This protocol was adapted as previously described^84^.

For mapping with LYVE-ef660, after 4 h following injection, images were obtained with an IVIS 2000 In Vivo Imaging System (Living Image Version 3.0) with a 1 -s exposure with the Cy5.5 excitation filter; animals were kept under anesthesia with 3% isoflurane during imaging.

### Ce3D tissue preparation and analysis

Clearing-enhanced 3D imaging of en bloc resected murine tongue and cervical tissues were performed as previously described^28,85^. Briefly, animals were fixed perfused with 8% methanol-free paraformaldehyde (EMS, Hatfield, PA). Following perfusion-fixation, en bloc resection of the tongue and cervical tissues was performed: the oral cavity was opened by extending incisions posteriorly from the oral commissure to the rami of the mandible, which were then transected. Next, the floor of mouth was dissected from the body of the mandible, which was subsequently liberated and removed from adjacent tissues. Blunt dissection was carried out circumferentially and inferiorly to liberate the anterior cervical tissues en bloc with the oral cavity soft tissues, extending in the anterior-posterior direction from deep to the dermis to the floor of the neck. Following resection, tissues were incubated overnight in 1:3 diluted cytofix in PBS (BD 554655) at 4 °C with agitation. The tissues were then washed for 24 h in PBS at 4 °C, which was changed twice. Tissues were then blocked at room temperature for 24 h in 1% normal mouse serum with 1% BSA and 0.3% Triton X-100 followed by staining with anti-LYVE-ef660 1:100 in blocking solution for 48 h at 37 °C with agitation and protected from light. Stained tissue was then washed with 0.2% Triton X-100 and 0.05% Thioglycerol in PBS for 24 h at 37 °C with agitation and protected from light; the washing solution was exchanged 3–4 times. Wash buffer was then evacuated, and tissues blotted prior to incubation with freshly prepared clearing solution—40% N-methylacetamide, 86% w/v Histodenz, 0.1% Triton X-100, and 0.5% 1-Thioglycerol in PBS—for 24 h with the exchange of clearing solution twice. Stained and cleared tissues were then mounted onto glass slides and placed into windows cut from silicone gaskets. Tissues seated within the windows cut from silicone gaskets were then bathed with clearing solution and coverslips applied. The Leica SP8 confocal was used to image Ce3D specimen; Leica (.lif) files were converted using the ImarisFileConverter 9.7.2 software and post-processing performed using Imaris Software 9.6.0.

### Cell lines and tissue culture

The 4MOSC1 syngeneic mouse HNSCC cells harboring a human tobacco-related mutanome and genomic landscape were developed and described for use in immunotherapy studies in our prior report^5,30^. MOC1 syngeneic mouse HNSCC cells derived from DMBA-induced oral tumors were generously provided by Dr. R. Uppaluri^86^. 293T cells (ATCC CRL-3216) were cultured in Dulbecco’s Modified Eagle’s Medium (DMEM) supplemented with 10% fetal bovine serum, 2 mM l-glutamine (ATCC 30-2214) and 1% antibiotic/antimycotic solution. 4MOSC1 cells were cultured in Defined Ketatinocyte-SFM medium supplemented with EGF Recombinant Mouse Protein (5 ng/ml), Cholera Toxin (50 pM) and 1% antibiotic/antimycotic solution. MOC1 cells were cultured in HyClone™ Iscove’s Modified Dulbecco’s Medium (IMDM; GE Healthcare Life sciences, South Logan, UT, USA, #sh30228.02)/HyClone™ Ham’s Nutrient Mixture F12 (GE Healthcare Life sciences# sh30026.01) at a 2:1 mixture with 5% fetal bovine serum, 1% antibiotic/antimycotic solution, 5 ng/mL EGF, 400 ng/mL hydrocortisone (Sigma-Aldrich, St Louis, MO, USA, #H0135), and 5 mg/mL insulin (Sigma-Aldrich, #I6634). All cells were cultured at 37 °C in the presence of 5% CO_2_.

#### Cloning of pLenti-eGFP-LucOS

The full-length coding sequence of LUC-OS flanked by attbB1/2 recombination site was amplified from the Lenti-LucOS (22777) using the LUC-OS-F (5^′^-GGGGACAAGTTTGTACAAAAAGCAGGCTTAATGGAAGACGCCAAAAACATA-3^′^) and LUC-OS-R (5^′^-GGGGACCACTTTGTACAAGAAAGCTGGGTTTTACAAGTCCTCttCAGAAAT-3^′^) primer. The purified PCR product was incorporated into the pDONR221 vector via a BP Reaction and subsequently introduced into the pLenti-CMV-GFP-DEST (19732) through an LR reaction.

#### Generation of stable GFP-Luc and eGFP-LucOS expressing 4MOSC1

For lentivirus production, 293T cells were plated in a poly-d-lysine–coated 15-cm dish and, 16 h later, transfected with 30 mg pHAGE PGK-GFP-IRES-LUC-W or pLenti-eGFP-LucOS, 3 mg VSV-G, 1.5 µg Tat1b, 1.5 µg Rev1b, and 1.5 µg Gag/Pol using 25.2 µL P3000 reagent and 25.2 µL of Lipofectamine 3000 transfection reagent, and media was refreshed 16 h post-transfection. At 48 and 72 h, virus-containing media was collected, filtered through a low protein binding filter unit (PVDF, 0.45 µm, Sigma-Aldrich), and stored at 4 °C for up to 5 days prior to use. Lentivirus suspension was concentrated using Lenti-X concentrator per manufacturer standardized protocol (Takara Bio). Subsequently, 4MOSC1 cells were plated in a collagen-coated six-well plate. At 16 h, seeded cells were transduced using 200 µL of concentrated virus in 2 mL keratinocyte-defined serum-free media and 4 µg/mL polybrene, and the plate was immediately centrifuged for 15 min at 450×*g*. GFP expression was validated by fluorescent microscopy and flow cytometry. Transduced 4MOSC1 cells were sorted by FACS for viability and GFP-positivity using a FACS-Aria Cell Sorter (BD Biosciences).

#### Primary OT-I T cells preparation

Naive OT-I CD8+ T cells and splenocytes were isolated from the spleen of OT-I mice. CD8+ T cells were isolated using a mouse CD8+ isolation kit (StemCell). T cells and splenocytes were cultured in RPMI 1640 (GlutaMAX) with 10% heat-inactivated FBS, 1 mM sodium pyruvate, 50 mM b-ME, 10 mM HEPES, and 1 3 MEM-NEAA, 100 U/ml penicillin and 100 mg/ml streptomycin (later referred to as complete T-cell culture media). Cytokines in T-cell culture media were added as indicated. To generate effector CD8+ T cells, OT-I splenocytes were cultured in complete T-cell media containing 1 nM SIINFEKL (OVA257-264) peptide followed by the addition of 100 IU/mL rhIL-2. Effector CD8+ T cells were ready for use after 2–4 days in co-culture. All cells cultures were incubated at 37 °C under 5% CO_2_.

#### Induced bone marrow dendritic cell (iDCs) and antigen priming

Bone marrow was isolated from or WT C57BL/6J mice for generation of iDCs^57,58^. Following erythrocyte lysis, the bone marrow cells were resuspended in complete DC medium (RMPI 1640 + 25 mM HEPES + 10% FBS, 1% l-glutamine, 1% 200 mM sodium pyruvate, 1% MEM-NEAA, 1% penicillin–streptomycin, 0.5% sodium bicarbonate, 0.01% 55 mM 2-mercaptoethanol) supplemented with rmGM-CSF (50 ng/mL) and rmFlt3-L (200 ng/mL) (Biolegend, San Diego, CA). The culture medium was changed on day 9 of culture. After 16 days of culturing, non-adherent cells and loosely adherent cells were harvested and gently washed by DPBS for subsequent labeling experiments or for T-cell co-culture experiments. In the iDC-OT-I CD8+ T-cell interactions experiments, iDCs were activated overnight with class C ODN 2395 (InvivoGen, San Diego, CA) and then cultured with or without indicated antigen peptides at 37 °C for 30 min. Non-adherent cells in the culture supernatant and loosely adherent cells were harvested and gently washed with DPBS for subsequent experiments. iDCs were cultured with or without indicated antigen peptides or tumor lysates (tumor cell/DC ratio = 10:1) and with indicated T cells (T cell/iDC ratio 1:2) in a complete T-cell medium prior to endpoint analysis as described below.

### TIL isolation and flow cytometry

Tumors were isolated, minced, and resuspended into the Tumor Dissociation Kit (Miltenyi Biotec, San Diego, CA) diluted into DMEM for subsequent processing with the gentleMACS Octo Dissociator, according to the manufacturer’s recommendations for tumor dissociation into a single-cell suspension. Digested tissues were then passed through 70-µm strainers to produce a single-cell suspension. Samples were washed with PBS and processed for live/dead cell discrimination using Zombie viability stains (Biolegend, San Diego, CA). Cell suspensions were then washed with cell staining buffer (Biolegend 420201) prior to cell surface staining, performed at the indicated antibody dilutions for 30 min at 4 °C, and protected from light. Cell surface staining was performed for 30 min at 4 °C with the following mouse antibodies: CD45 (30-F11) (1:100), CD3 (17A2) (1:200), CD8a (53-6.7) (1:100), CD4 (RM4-4) (1:100), Slamf6 (330AJ) (1:100), PD-1 (29 F.1A12) (1:100), CD44 (IM7) (1:100), CD19 (6D5) (1:100), CXCR3 (S18001A) (1:100), Tim3 (RMT3-23) (1:100), NK1.1 (PK136) (1:100), CD69 (H1.2F3) (1:100), CD62L (MEL-14) (1:100), BST2 (129C1) (1:100), Ly6C (HK1.4) (1:100), CD11b (M1/70) (1:100), CD11c (N418) (1:100), Siglec H (551) (1:100), XCR1 (ZET) (1:100), CD64 (X54-5/7.1) (1:100), CD103 (2E7) (1:100), SIRPa (P84) (1:100), MHCII (M5/114.15.2) (1:200), CD80 (16-10A1) (1:100), CD86 (GL-1) (1:100), Ep-CAM (G8.8) (1:100) and H-2Kb-SIINFEKL (25-D1.16) (1:100). Stained cells were washed and then fixed with BD cytofix for 20 min at 4 °C, protected from light. In the case of intracellular staining, permeabilization was then performed by incubating with fixation-permeabilization buffer (ThermoFisher 88-8824-00) according to the manufacturer’s recommendations prior to staining with intracellular targeted antibodies at the indicated dilutions in permeabilization buffer for 30 min at 4 °C and protected from light. Intracellular antibodies used: IL-2 (JES6-5H4) (1:100) and IFNγ (XMG1.2) (1:100). Cells were washed twice with permeabilization buffer and subsequently with cell staining buffer. For antigen-specific T cell tetramer staining, either the Flex-T™ Biotin H-2 K(b) OVA Monomer (Biolegend 280051) paired with PE-streptavidin or APC-streptavidin (Biogened 405203 or 405207, respectively) or the H-2Kb MuLV p15E Tetramer-KSPWFTTL-PE (MBL TB-M507-1) was used according to manufacturers’ instructions and as previously described^42^. Samples were acquired using a BD LSRII Fortessa. Downstream analysis was performed using TreeStar FlowJo, version 10.6.2. Representative flow cytometry gating strategies are detailed in the supplementary figures.

### Mass cytometry (CyTOF)

Tissues were incubated for 15 min at 37 °C and mechanically digested using the gentle MACs Octo Dissociator. Digested samples were then passed through a 100-μm strainer to acquire a single cell. For viability staining, cells were washed in PBS and stained with Cell-ID Cisplatin (DVS Sciences) to a final concentration of 5 μM for 5 min at room temperature. Cisplatin was quenched when cells were washed and stained with the antibody cocktail. Antibodies were prepared in Maxpar cell staining buffer (PBS with 2 mM EDTA, 0.1% BSA, 0.05% NaN_3_) and incubated with cells for 15 min at room temperature. Cells were stained with the following antibodies from Fluidigm: B220 (RA3-6B2), CD117(2B8), CD11c (N418), CD25 (3C7), CD4 (RM4-5), CD45 (30-F11), CD8a (53-6.7), MHCII (M5/114.15.2), NKP46 (29A1.4), CD169 (3D6.112), CD206 (C068C2), and TCRb (H57-597); or from BioLegend: CD103 (2E7), CD115 (AFS98), CD11b (M1/70), CD19 (6D5), CD3 (145-2C11), CD64 (X54-5/7.1), F4/80 (BM8), FR4 (TH6), Ly6C (HK1.4), Ly6G (1A8), and NK1.1 (PK136); or from eBioscience (ThermoFisher Scientific): Siglec-F (1RNM44N). All antibodies were used at a 1:100 dilution. After staining, cells were washed and fixed with 1.6% formaldehyde (FA) for 10 min at room temperature. For cell identification, cells were washed in staining buffers and stained with DNA intercalator (Fluidigm) containing natural abundance Iridium (191Ir and 193Ir) prepared to a final concentration of 125 nM. Cells were washed with staining buffer and pelleted. Before acquiring, cells were resuspended in 0.1× dilution of EQ Four Element Calibration beads (Fluidigm) and filtered through a 35-μm nylon mesh filter. Cells were acquired on a Helios CyTOF Mass Cytometer (Fluidigm) at an event rate of 200 events/second or less. Data were normalized using Matlab-based normalization software based on the EQ bead removal. To detect clusters of cells with a similar expression of surface markers in CyTOF, single cells were gated and clustered using unsupervised dimensionality reduction algorithm optimal t-Distributed Stochastic Neighbor Embedding (opt-SNE) algorithm in OMIQ data analysis software 2022 (www.omiq.ai), 530 iterations, Perplexity 30, and Theta 0.5.

### Tissue analysis

#### Histology

Tissue samples were fixed in zinc formalin fixative (Sigma-Aldrich) and sent to HistoServ, Inc. (Germantown, MD) for embedding, sectioning, and H&E staining. Histology samples were analyzed using QuPath 0.2.3, an open-source quantitative Pathology & Bioimage Analysis software (Edinburgh, UK). Immunohistochemistry on formalin-fixed paraffin-embedded lymph node samples or tumor samples was performed using anti-wide spectrum cytokeratin antibody (Abcam, ab9377, 1:200 dilution, overnight at 4 °C), CD8 (Abcam ab22378, 1:400 dilution overnight at 4 °C) or CD4 (ab183685, 1:400 dilution overnight at 4 °C). Tissues were then counterstained with biotinylated anti-rabbit secondary (Vector Labs, BA-1000, 1:400 dilution, 30 min at room temperature) or Goat Anti-Rat IgG H&L (HRP) (ab205720, 1:400, 30 min at room temperature). The protocol utilized is described in detail in ref. ^87^, with the following modifications (1) antigen retrieval was performed using low pH IHC Ag Retrieval Solution (ThermoFisher, 00-4955-58) and subjected to heat using a steamer for 40 min, and (2) Bloxall Blocking Solution (Vector Labs, SP-6000, 20-min incubation, room temperature) was used to inactivate endogenous peroxidases. Slides were processed with either the ABC reagent (Vector Laboratories, # PK-6100) or the DAB substrate kit (Vector Laboratories, # SK-4105). Slides were scanned using a Zeiss Axioscan Z1 slide scanner equipped with a ×20/0.8 NA objective. All image analyses were performed using the QuPath software to perform pixel classification of stained cells.

#### Multiplex immunofluorescence

In all, 4-µm sections were cut on a Microm HM355S microtome (ThermoScientific) and floated onto plus-slides (Cardinal ColorFrost). Slides were allowed to dry at RT overnight. Slides were placed onto a staining rack in the Leica autostainer and deparaffinized (xylene—4 min; 100% ethanol—2 min; 95% ethanol—1 min; 70% ethanol—1 min; water). Slides underwent antigen retrieval in AR9 Buffer (PerkinElmer, AR900250ML) for 1 min (100% Power) and 10 min (10% Power) in a microwave. Slides were then treated with PeroxAbolish (Biocare Medical) for 20 min to reduce endogenous peroxidase activity. Slides were rinsed with H_2_O and TBS-T and blocked with goat serum (Vector Labs) for 20 min. Rabbit anti-CD11c (D1V9Y, Cell Signaling Technology, 1:250) was diluted in Renaissance antibody diluent (Biocare Medical), added to the slide, and incubated for 45 min on an orbital shaker at RT. After washing in TBS-T, anti-rabbit secondary HRP (Vector Labs, MP-7451-15) was added for 15 min RT, and subsequently washed with TBS-T. Slides were incubated with Opal620 reagent (FisherScientific, NC1612059) at 1:250 dilution in Amplification plus buffer (PerkinElmer, NEL791001KT) for 10 min at RT and washed in TBS-T and H_2_O. For the second cycle, slides were treated with PeroxAbolish for 20 min to eliminate peroxidase activity. The slides were then stained with rat anti-CD8 (4SM15, ThermoFisher, 14-0808-82, 1:1750), washed in TBS-T, anti-rat secondary HRP (Vector Labs, MP-7444-15) added, washed in TBS-T, and incubated with Opal520 reagent (FisherScientific, NC1601877) at 1:150 for 10 min. For the third cycle, slides underwent antibody stripping in Rodent Decloaker (Biocare Medical, RD913) for 1 min (100% Power) and 10 min (10% Power) in a microwave, blocked with goat serum (Vector Labs) for 20 min RT, stained with rabbit anti-CD103 (Abcam, ab224202, 1:1500) for 45 min RT, anti-rabbit secondary HRP for 15 min RT, and Opal570 reagent (FisherScientific, NC1601878) at 1:200 for 10 min RT. For the fourth cycle, slides underwent antibody stripping in Rodent Decloaker (Biocare Medical, RD913) for 1 min (100% Power) and 10 min (10% Power) in a microwave, blocked with goat serum (Vector Labs) for 20 min RT, stained with rabbit anti-CD3 antibodies (SP7, Abcam, ab16669, 1:75) for 45 min RT, anti-rabbit secondary HRP for 15 min RT, and Opal690 reagent (FisherScientific, NC1605064) at 1:100 for 10 min RT. After washes in TBS-T, DAPI (Life Technologies, D1306, 1 mg/mL stock, 1:500 in PBS) was added to slides for 10 min at RT. Slides were rinsed with TBS-T and H_2_O and coverslipped with VectaShield Hard Mount (Vector Labs). Slides were imaged at both ×10 and ×20 using the Vectra 3 Polaris and Vectra imaging software (Akoya Biosciences). Acquired qpTIFF images from the Vectra Polaris system were imported into QuPath analysis software^88^ and the whole image analysis was performed using the pixel classification algorithm. Pixel classification was employed to quantify the high density of cells in the lymph node.

### Chemokine array

Tumors and tumor-draining lymph nodes were isolated and tissue homogenate in lysis buffer (20 mM Tris HCl pH 7.5, 0.5% Tween 20, 150 mM NaCl) supplemented with protease inhibitors. Mouse Chemokine Array 44-Plex (MD44) was run by EVE Technologies (Calgary, AB, Canada).

### In vivo mouse models and analysis

All the animal studies using HNSCC tumor xenografts and orthotropic implantation studies were approved by the University of California San Diego (UCSD) Institutional Animal Care and Use Committee (IACUC), with protocol ASP #S15195; and all experiments adhere with all relevant ethical regulations for animal testing and research. All mice were obtained from Charles River Laboratories (Worcester, MA). Mice at UCSD Moores Cancer Center are housed in individually ventilated and micro-isolator cages supplied with acidified water and fed 5053 Irradiated Picolab Rodent Diet 20. The temperature for laboratory mice in this facility is mandated to be between 18 and 23 °C with 40–60% humidity. The vivarium is maintained in a 12-h light/dark cycle. All personnel were required to wear scrubs and/or lab coat, mask, hair net, dedicated shoes, and disposable gloves upon entering the animal rooms. WT C57Bl/6 mice were obtained from Charles River Laboratories (Worcester, MA). C57Bl/6 OT-1 (Tg-TcraTcrb-1100Mjb/J), IFNAR KO (*Ifnar1*^*tm1.2Ees*^/J), and BATF3 KO (Batf3tm1Kmm/J) animals were obtained from The Jackson Laboratory (Bar Harbor, ME). C57Bl/6 XCR1^DTRVenus^ animals were a kind gift from Dr. Tsuneyasu Kaisho (Wakayama Medical University). Depletion of DTR^venus^-expressing cells was achieved with intraperitoneal injection of Diptheria toxin (DT 322326, Millipore Sigma) at a dose of 25 ng/g body weight every 3 days^59^.

#### Orthotopic tumor modeling

For orthotopic implantation, 4MOSC1 cells were transplanted (1 million per tumor) into the oral cavity of female C57Bl/6 mice (4–6 weeks of age), either into the tongue or buccal mucosa. MOC1 cells were transplanted into tongue (1 million per tumor) of female C57Bl/6 mice (4–6 weeks of age). For drug treatment, the mice were treated by intraperitoneal injection (ip), CTLA-4 antibody, CLTA-4 + IA8 antibody, or PD-1 antibody. The mice were sacrificed at the indicated time points (or when mice succumbed to disease— tongue tumors >8 mm in greatest diameter or ulcerated; buccal tumors >1 cm in greatest dimension or ulcerated—as determined by the ASP guidelines) and tissues were isolated for histological and immunohistochemical evaluation or flow cytometric analysis. The maximum tumor size/burden permitted in accordance with our institutional review board was not exceeded.

#### Surgery

All the animal surgery procedures were approved by the University of California San Diego Institutional Animal Care and Use Committee (IACUC), with protocol #S15195. Mice were dosed with 0.1 mg/kg buprenorphine every 12 h as needed for pain. Neck dissection was performed as described above. Briefly, anesthetized animals were positioned and draped and prepped in sterile fashion. Under ×8 operative microscopy, the skin was incised sharply in the midline with straight microscissors and skin flaps were bluntly elevated laterally to broadly expose the cervical space spanning from the angle of the mandible bilaterally to the clavicles. Superficial lymphatic basins were encountered immediately deep to the dermis and adjacent the superolateral aspects of the submandibular glands and were liberated with blunt dissection and handheld monopolar cautery from surrounding tissues. Reflecting the submandibular glands and superficial lymphatic basins laterally revealed the jugular venus plexus and deep lymphatic basins nested within the jugular vascular confluence and atop the floor of the neck. Deep lymphatic tissues were resected after blunt dissection to liberate them from surrounding tissues. After resection, hemostasis was confirmed or achieved with cautery. Native tissues were repositioned and the wound was closed in a single layer with 5-0 simple interrupted vicryl sutures. Animals were placed under a heating lamp in a recovery space and observed until fully conscious. For the sham surgery group, mice were anesthetized, and skin flaps were raised with care to not disturb underlying lymphatic channels; no tissues were resected in sham group animals.

#### Subtotal primary tumor resection

For the subtotal primary tumor ablation, buccal tumor-bearing animals were positioned and draped in a sterile fashion. Under loupe-assisted magnification, the a small 0.1–0.2 mm skin incision overlying tumors was introduced and blunt dissection with microscissors was used to expose the tumor. Following exposure, gross tumor specimen was resected with care to ensure that a 2 × 2 mm gross + margin was left in situ without disturbing the relationships to surrounding tissues or vasculature.

#### Radiation

The dedicated small animal radiotherapy planning system SmART-Plan (version 1.3.1, Precision X-ray, North Branford, CT) was used to create, evaluate, and deliver irradiation^89^. Animals were anesthetized with isoflourane and positioned within the SmART machine, secured to the stage. A spiral CT scan with 1 mm cuts of the neck was obtained and cervical lymphatics delineated as the planning target volume. A 5 mm collimator was installed, and two static parallel opposed beams linked to the irradiator isocenter were used to deliver homogenous single fraction doses to the planned target volume.

#### Imaging

For IVIS imaging, 4MOSC1 cells expressing luciferase were injected into the buccal mucosa of C57Bl/6 mice. After 3 days, bioluminescence was assessed twice weekly by bioluminescence images captured using the In Vivo Imaging System (IVIS) Spectrum (PerkinElmer). Mice received an intraperitoneal injection of 200 mg/kg d-luciferin firefly potassium salt diluted in PBS 15 min before imaging (GoldBio). IVIS data were collected as a correlate to tumor kinetics measurements and without quantification, as shown in the figures.

#### Local tumor-draining lymph node injection

Tumor-draining lymph nodes were exposed after skin incision and careful soft tissue dissection. Once delivered from the wound, the tumor-draining lymph node was gently grasped with smooth pickups in an atraumatic fashion, and a 10 μL Hamilton 1800 Series Gastight Syringe loaded with a 31-gauge, 0.5 in, number 4 45-degree needle was gently introduced. The bevel of the needle was visualized to enter the tdLN under otomicroscopic magnification and the payload was delivered: either 1 μL 5 mg/mL MAR1-5A3, repeated on days 1, 3, 5 & 7, or 1 μL 1 mg/mL diphtheria toxin, repeated on days 3, 6, and 9. After confirmation of payload delivery with no retrograde spillage, the needle was removed, tdLN replaced into the neck with soft tissue overlayed and neck closed with simple interrupted 5-0 vicryl sutures.

### Enzyme-linked immunosorbent assay (ELISA)

The concentrations of cytokines in tumor-draining lymph node suspensions were measured using mouse IFN-β (439407, BioLegend) enzyme-linked immunosorbent assay (ELISA) kits according to the manufacturer’s instructions.

### Enzyme-linked immunosorbent spot assay (ELISpot)

Mice were euthanized and the regional lymphatics and spleens were harvested and placed into RPMI with 10% fetal bovine serum (FBS) and 1% (v/v) penicillin/streptomycin (R10 media). Lymph nodes and spleens were pummeled and passed through a 70-μm cell strainer to achieve single-cell suspensions. Suspensions were layered under Lympholyte Cell Separation Media (Cedarlane) and centrifuged (1250×*g*, 30 min, 24 °C). Pellets were resuspended at 4 × 10^6^ cells/ml in R10 medium and plated at 2 × 10^5^ cells/well on 96-well MultiScreen-IP filter plates, pre-coated with anti-mouse IFNγ mAb AN18 (Mabtech). Control, concanavalin A (5 μg/mL), or SIINFEKL-pulsed BMDCs were co-cultured at 2 × 10^4^ cells/well. After incubation in humidified 5% CO_2_ at 37 °C for 20 h, cells were removed by washing and incubated with 1 μg/ml biotinylated secondary anti-IFNγ antibody R4-6A2 (Mabtech). After 2 h at room temperature, plates were washed with PBS and incubated in streptavidin-HRP for 1 h. Plates were developed using TMB substrate and halted after the formation of distinct spots. Interferon-gamma-positive spots were imaged, analyzed, and counted using the AID ELISpot reader and the AID EliSpot/FluoroSpot Software V7.0 (AID).

### RNA sequencing and analysis

#### Blood collection and RNA isolation

Blood was collected with cheek bleeds into BD Microtainer capillary blood collection tubes (365974) to a volume of at least 100 μL per animal. Care was taken to remove clots from samples and 100 μL of each sample was transferred into microcentrifuge tubes containing 1 mL TRIzol. RNA was then isolated using Qiagen RNeasy^®^ Mini Columns (74004; Qiagen, Germantown, MD) according to the manufacturer’s recommendations and including an on-column DNase I digestion. Yield and integrity of RNA was confirmed by reading absorbance at 260, 280, and 230 nm using a NanoDrop ND-1000 (NanoDrop Technologies; ThermoFisher Scientific, Inc., Wilmington, DE, USA) and with the Agilent 2200 Tapestation (Agilent Technologies, Inc.). Library preparation and paired-end 150 bp (PE150, Illumina) RNA sequencing was performed by Novogene (Novogene Corporation, Sacramento, USA).

#### Alignment/differential expression

Paired-end reads were aligned using STAR v2.7.9 using default options. STAR index was created using the GRCm39 primary genome FASTA and annotation files. The resulting BAM files were sorted by name using samtools v1.7 then gene counts were quantified using HTSeq-count v0.13.5. Pairwise differential expression was calculated and PCA plots were created using DESeq2 v1.32.0.

#### GSEA/GO

Gene set enrichment analysis was conducted using the GSEAPreranked v7.2.4 module on the GenePattern public server, gsea-msigdb.org, with 10,000 permutations, and the genes mapped and collapsed to standard mouse symbols using the MSigDB mapping file version v7.4^63,64^. The Gene Ontology (Biological Processes) and ImmunesigDB gene set collections were used^65^. The ranked list of genes was created using the log_2_-fold change (FC) calculated by DESeq2 for the comparison of αCTLA-4-treated animals receiving either early or late neck dissection. For this analysis, genes more highly expressed in late relative to early neck dissection are at the top of the ranked list. Gene ontology (GO) analysis was performed through the GeneOntology.org website using the top significant (log_2_FC > 1, *P* value < 0.05) upregulated genes in the samples from the late neck dissection group.

### Statistics and reproducibility

Data analysis was performed with GraphPad Prism version 9 for Mac. The differences between experimental groups were analyzed using independent *t* tests, one- or two-way ANOVA with multiple comparisons, fisher’s exact test, DESeq2, Log_2_FC *P* < 0.05, or simple linear regression analysis as indicated. Survival analysis was performed using the Kaplan–Meier method and log-rank tests. The asterisks in each figure denote statistical significance, or ns for non-significant ****P < 0.0001. All the data are reported as mean ± SEM (standard error of the mean). For all experiments, each experiment was independently repeated at least twice with similar results.

### Reporting summary

Further information on research design is available in the Nature Research Reporting Summary linked to this article.

## Supplementary information

Supplementary Information

Reporting Summary

## Data Availability

The RNA sequencing data generated in this study have been deposited in the Gene Expression Omnibus database under accession code GSE197250. The remaining data are available within the Article, Supplementary Information or Source Data file. Source data are provided with this paper.

## Source data

Source Data
