# Supplementary material for: Lymphatic-preserving treatment sequencing with immune checkpoint inhibition unleashes cDC1-dependent antitumor immunity in HNSCC
Source: Nat Commun. 2022 Jul 25;13:4298. doi: 10.1038/s41467-022-31941-w (PMC9314425; doi:10.1038/s41467-022-31941-w)
Supplement: Supplementary file 1 — Supplementary Information [file 41467_2022_31941_MOESM1_ESM.pdf]

Supplementary Figure 1: Cervical Lymphatic Mapping & Neck Dissection Model

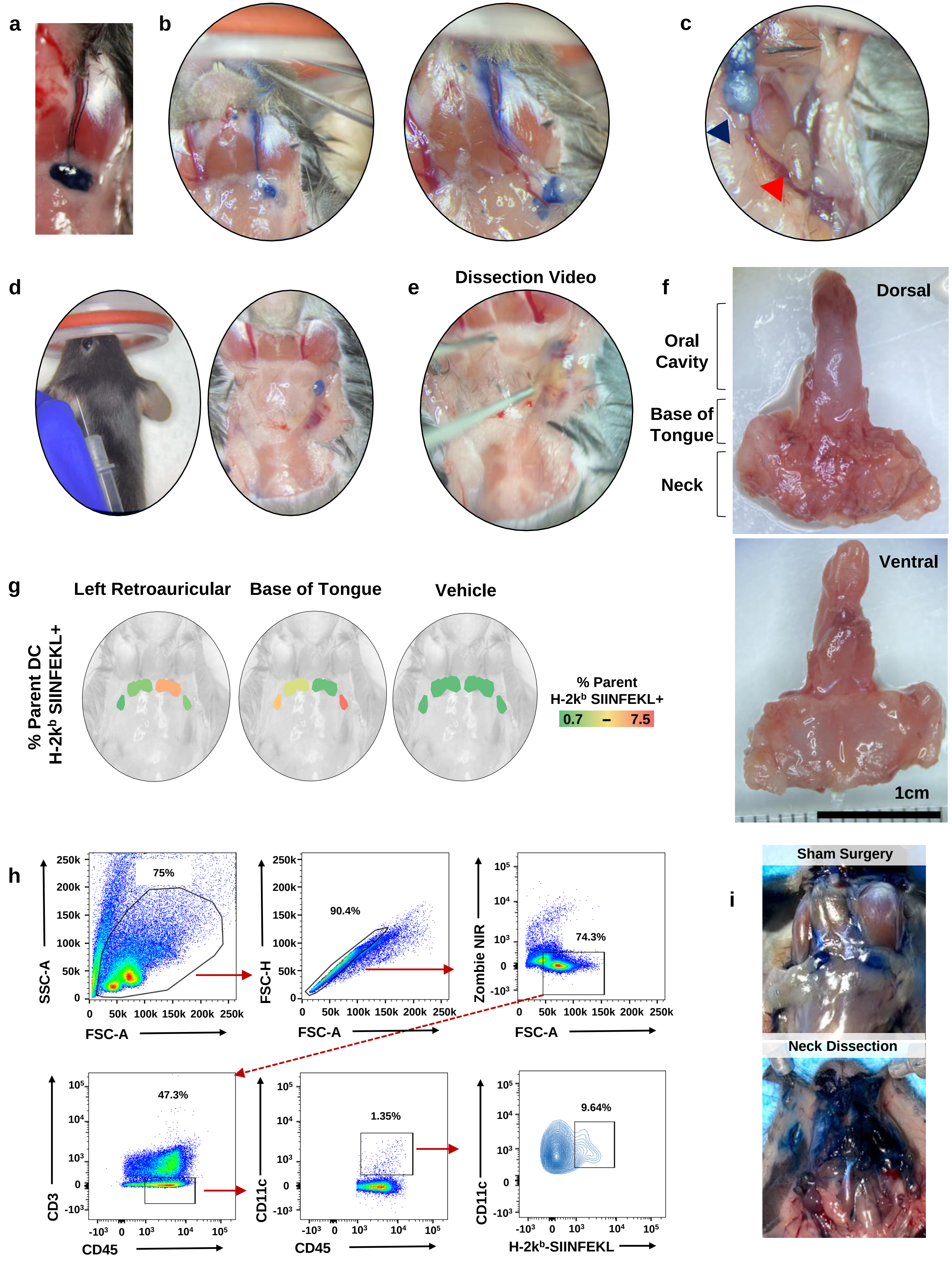

Supplementary Fig. 1: Cervical Lymphatic Mapping & Neck Dissection Model

(A-B) Illustrative photographs depicting the lymphatic drainage pathways filled with 5% Evans Blue Dye that collects into draining lymph nodes after injection into the left buccal mucosa.

(C) Illustrative photograph depicting the collection of 5% Evans Blue Dye into superficial lymph nodes (blue arrowhead) but not deeper lymph nodes (red arrowhead) after injection into the tongue.

(D-E) Illustrative photographs and (E) video depicting anatomic lymphatic mapping following injection of 5% Evans Blue dye into the retroauricular subcutaneous space, and visualization of dye collection into draining lymphatic basins.

(F) Representative specimen used in clearing-enhanced 3D imaging in Fig 1B.

(G) Functional mapping of lymphatic drainage basins following injection of SIINFEKL peptide with CpG adjuvant into the left retroauricular space or base of tongue. Depicted are lymphatic basins, overlayed with heatmaps, to indicate the % of CD11c+ H-2k^b SIINFEKL+ cells isolated, stained and analyzed by flow cytometry 12 hours following injection.

(H) Gating strategy to identify CD11c+ H-2k^b SIINFEKL+ cells in lymph nodes following injection of SIINFEKL peptide into subsites of interest; associated with Fig 1C & Supp Fig 1G.

(I) Illustrative photographs depicting the drainage patters of 5% Evans blue dye following injection into the tongue in animals subjected to either a sham surgery (left) or neck dissection (right).

Supplementary Figure 2: Draining Lymphatic Basins are Required for Tumor Response to Immune Checkpoint Inhibition

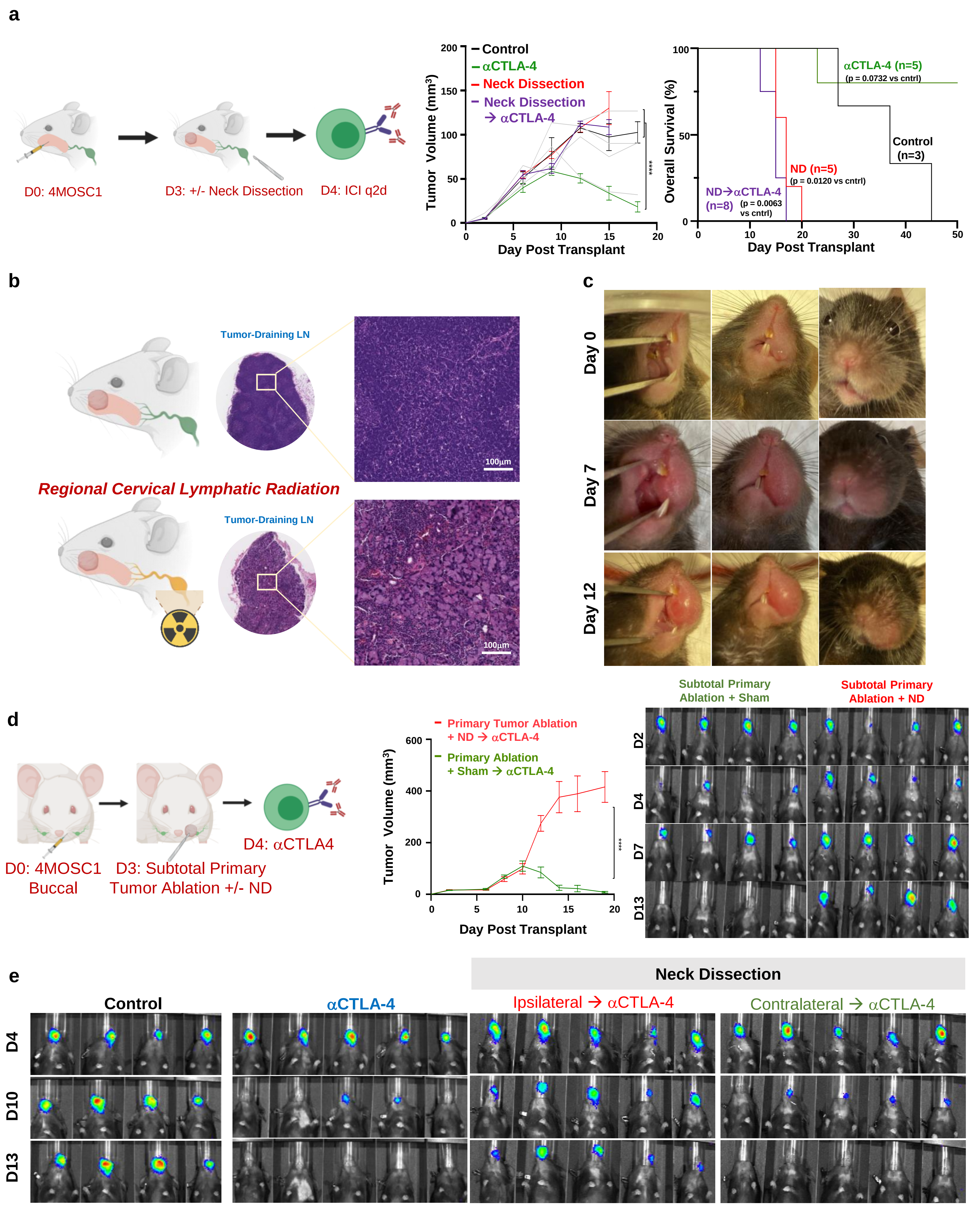

Supplementary Fig. 2: Draining Lymphatic Basins are Required for Tumor Response to Immune Checkpoint Inhibition

(A) Cartoon depicting the experimental schema. 10^6 4MOSC1 tumor cells were orthotopically transplanted into the tongues, after which animals were randomized at day 3 to receive neck dissection alone or neck dissection followed by αCTLA-4 treatment; (**Middle**) tumor growth kinetics and (**Right**) overall survival to day 50 are shown (n = 4-8/group).

(B) Representative H&E images of tumor draining lymph nodes from 4MOSC1 tongue tumor-bearing animals (**Top**) without radiation therapy to the cervical lymphatics or (**Bottom**) after a single 18Gy dose of radiation therapy, directed to the tumor draining lymphatics (representative of n=5 tdLNs/treatment group).

(C) Representative images following injection of 4MOSC1 cells into the buccal mucosa, detailing the development of tumors over 12 days.

(D) (**Left**) Cartoon depicting the experimental schema. 10^6 4MOSC1-LucOS tumor cells were orthotopically transplanted into the buccal mucosa of recipient animals. Following the development of conspicuous tumors (day 3), animals received subtotal primary tumor ablation and were randomized to receive sham versus neck dissection and treatment with αCTLA-4 (**Right**) tumor growth kinetics and in vivo imaging (IVIS 2000) on days 2, 4, 7, and 13 for the indicated IO treatment regimen (n = 5 animals/group).

(E) In vivo imaging (IVIS 2000) of 4MOSC1-LucOS buccal tumor bearing animals after treatment with αCTLA-4 ICI monotherapy, or therapy following ipsilateral or contralateral neck dissection, obtained at day 4, 10, and 13 (n = 4-5/group).

The differences between experimental groups were analyzed using simple linear regression analysis (Supp Fig 2A & D) and survival analysis was performed using the Kaplan–Meier method and log-rank tests (Supp Fig 2A) . All data represent averages ± SEM, excepted where indicated. **** = $p < 0.0001$, ns = not statistically significant. Source data are provided as a Source Data file.

Supplementary Figure 3: Regional Tumor-Draining Lymphatics Coordinate Antigen-Specific CD8-Driven Immunity in the Tumor Microenvironment

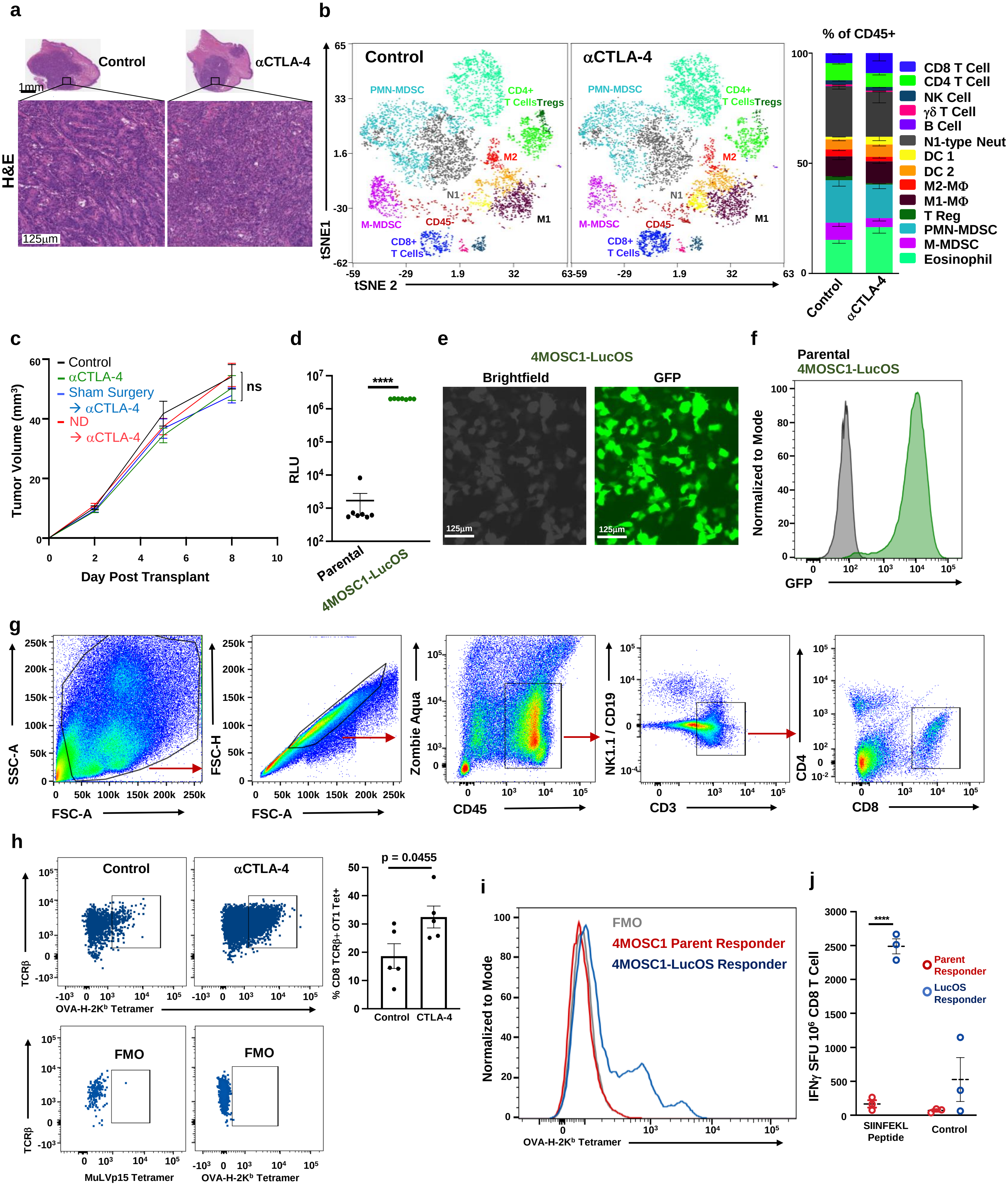

Supplementary Fig. 3: Regional Tumor-Draining Lymphatics Coordinate Antigen-Specific CD8-Driven Immunity in the Tumor Microenvironment

(A) Representative immunohistochemical images of 4MOSC1 tongue tumors from control or αCTLA-4 treated animals harvested at day 10. Shown are whole tumor sections and representative high-power H&E (representative of n=5 tumors/group).

(B) (**Left**) Representative tSNE plots shown from a time-of-flight mass cytometry (CyTOF) experiment comparing 4MOSC1 tongue tumors from control or αCTLA-4 treated animals, harvested at day 10; (**Right**) Quantification of selected populations identified in the TIME of the aforementioned groups, indicated by color and depicted as percentage of CD45+ cells (n = 3 independent samples / group).

(C) Tumor growth kinetics of tumors harvested for CyTOF analysis, demonstrating equivalent tumor sizes across treatment cohorts at time of sample collection (n = 5/group).

(D-F) Expression of GFP in the novel 4MOSC1 pLenti-GFP-LucOS model, as indicated by quantified RLU (D; n = 7 independent samples/group), representative images (E), and flow cytometry (F).

(G) Gating strategy to identify CD8+ T cells to identify those with tetramer positive TCRs; associated with Fig 3G-H, Fig 4N & Supp Fig 3H.

(H) (**Top**) Representative flow cytometry plots and quantification identifying TCRb+ OVA-H-2kb Tetramer+ CD8+ T cells from control and αCTLA-4 treated 4MOSC1-LucOS tongue tumor bearing animals harvested at day 10 with quantification shown (n = 5/group) (**Bottom**) Flow cytometry plots of FMO gates for tetramer staining of MuLVp15 and OVA-H-2kb model antigens.

(I) Representative overlayed histogram showing the expression of OVA-H-2K^b tetramer+ among CD8 T cell populations harvested from the cervical lymph nodes and spleens of either 4MOSC1 Parent or LucOS complete responders to aCTLA-4 therapy > 365 days after tumor clearance.

(J) IFNg SFU of CD8 T cells from Supplementary Fig. 3I after challenge with SIINFEKL-loaded induced bone marrow dendritic cells (n = 3).

The differences between experimental groups were analyzed using independent, two-sided student *t*-tests (Supp Fig 3D, H, J) or using simple linear regression analysis (Supp Fig3C). All data represent averages ± SEM, excepted where indicated. **** = $p < 0.0001$, ns = not statistically significant. Source data are provided as a Source Data file.

Supplementary Figure 4: Tumor Draining Lymphatics Harbor a Population of Conventional Type-I Dendritic Cells Critical for the Response to ICI

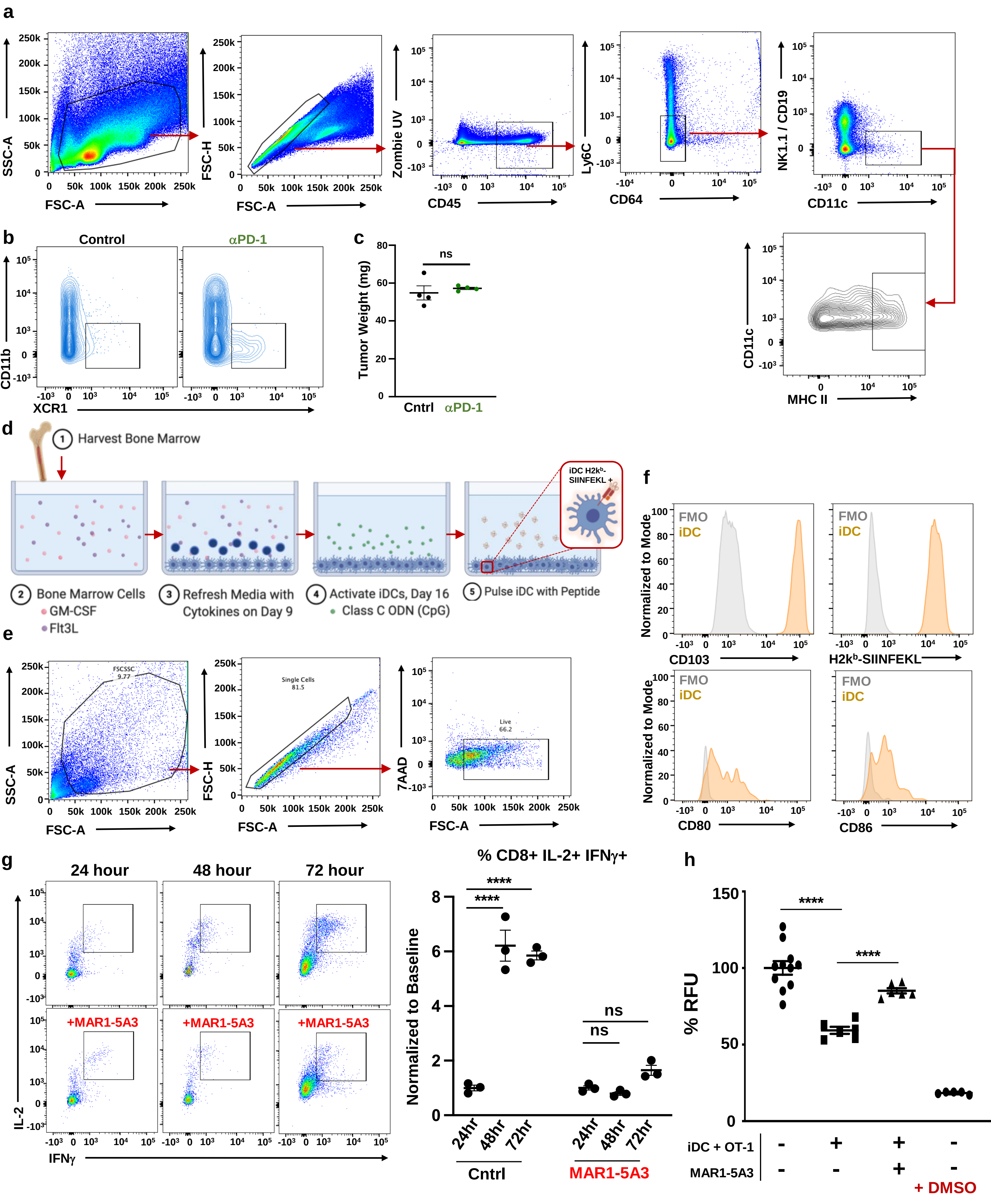

Supplementary Figure 4: Tumor Draining Lymphatics Harbor a Population of Conventional Type-I Dendritic Cells Critical for the Response to ICI

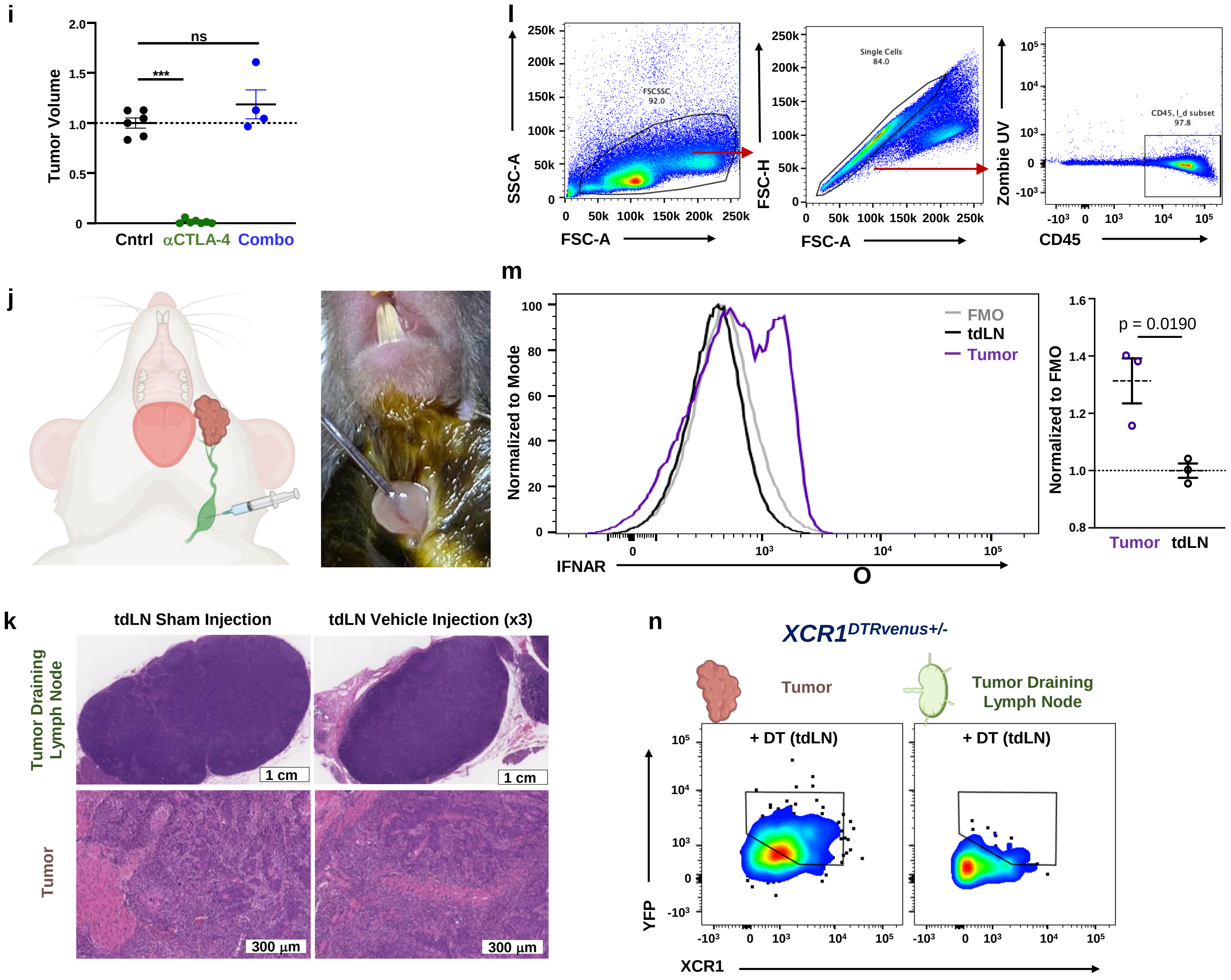

Supplementary Fig. 4: Tumor Draining Lymphatics Harbor a Population of Conventional Type-I Dendritic Cells Critical for the Response to ICI

(A) Gating strategy to identify dendritic cell populations in the tumor draining lymph nodes of 4MOSC1-tongue tumor bearing animals; associated with Fig 4C-D & Supp Fig 4B-C.

(B) Representative flow cytometry plots identifying cDC1s (Ly6c-CD64-CD19-NK-CD11c+MHCIIhi CD11b-XCR1+) from the tdLNs of control or αPD-1 treated 4MOSC1 tongue tumor bearing animals, harvested at day 10 after transplant.

(C) Tumor weight of control or αPD-1 treated 4MOSC1 tongue tumor bearing animals, harvested at day 10 after transplant (n = 4 independent samples /group).

(D) Cartoon depicting method of developing induced cDC1s (iDC) from bone marrow. Briefly, bone marrow was harvested from mouse femur and cultured with GM-CSF and Flt3L for 16 days, with cytokines refreshed on day 9. iDCs were activated with TLR9 agonist CpG, then pulsed with peptide to allow for cross-presentation of antigen.

(E) Gating strategy to identify iDCs for subsequent profiling as in Supplementary Fig 4F.

(F) Representative flow plots comparing iDC versus FMO expression of SIINFEKL peptide and co-stimulation markers after activation and pulse with H2k^{b}-SIINFEKL.

(G) Representative flow plots (**Left)** and quantification (**Right**) of IL-2 and IFNg expression in OT-1+ T cells cultured with SIINFEKL+ iDCs, with or without interferon blockade with MAR1-5A3; associated with Supp Fig 4G-H (n = 3 independent samples/group).

(H) Cytotoxicity assay comparing efficacy of antigen-specific killing of CD8+ T cells primed by iDCs, with or without interferon blockade with MAR1-5A3. The Y-axis indicates percent viability, normalized to control, of target cells expressing OVA following co-culture with OT-1 T cells activated by iDCs in vitro (control n=11; iDC+OT-1 n=6; iDC+OT-1+MAR1-5A3 n=6; DMSO n=5).

(I) 4MOSC1 tongue tumor normalized volume at day 16 following treatment with aCTLA-4 +/- MAR1-5A3, associated with Fig. 4J (n = 6, dead animals omitted).

(J) (**Left**) Cartoon schema for local injection into tumor draining lymph node; (**Right**) Representative photograph of local injection into the tumor draining lymph node in a 4MOSC1 buccal-tumor bearing animal.

(K) Representative H&E images of (**Top**) tdLNs and (**Bottom**) buccal tumors following serial sham or vehicle microinjections into the tdLN (representative of n=4 tdLNs and tumors/treatment group).

(L) Gating strategy to identify IFNAR+ CD45+ cells, as represented in Supplementary Fig. 4M

(M) (**Left**) Representative histogram overlay of IFNAR+ CD45+ populations, normalized to mode, with (**Right**) quantification (n = 3 individual samples/group).

(N) Representative flow cytometry plots from tumor or tdLN after local injection of diphtheria toxin into the tdLNs of 4MOSC1 buccal tumor bearing *XCR1*$^{DTRVenus+/-}$ GEMM animals.

The differences between experimental groups were analyzed using independent, two-sided student *t*-tests (Supp Fig 4C, I, M) or two-way anova with multiple comparisons (Supp Fig 4G-H). All data represent averages ± SEM, excepted where indicated. **** = p < 0.0001, ns = not statistically significant. Source data are provided as a Source Data file.

Supplementary Figure 5: Rational IO Treatment-Sequencing Drives Primary Tumor Treatment Responses and Immunosurveillance to Protect Against Locoregional Nodal Metastasis

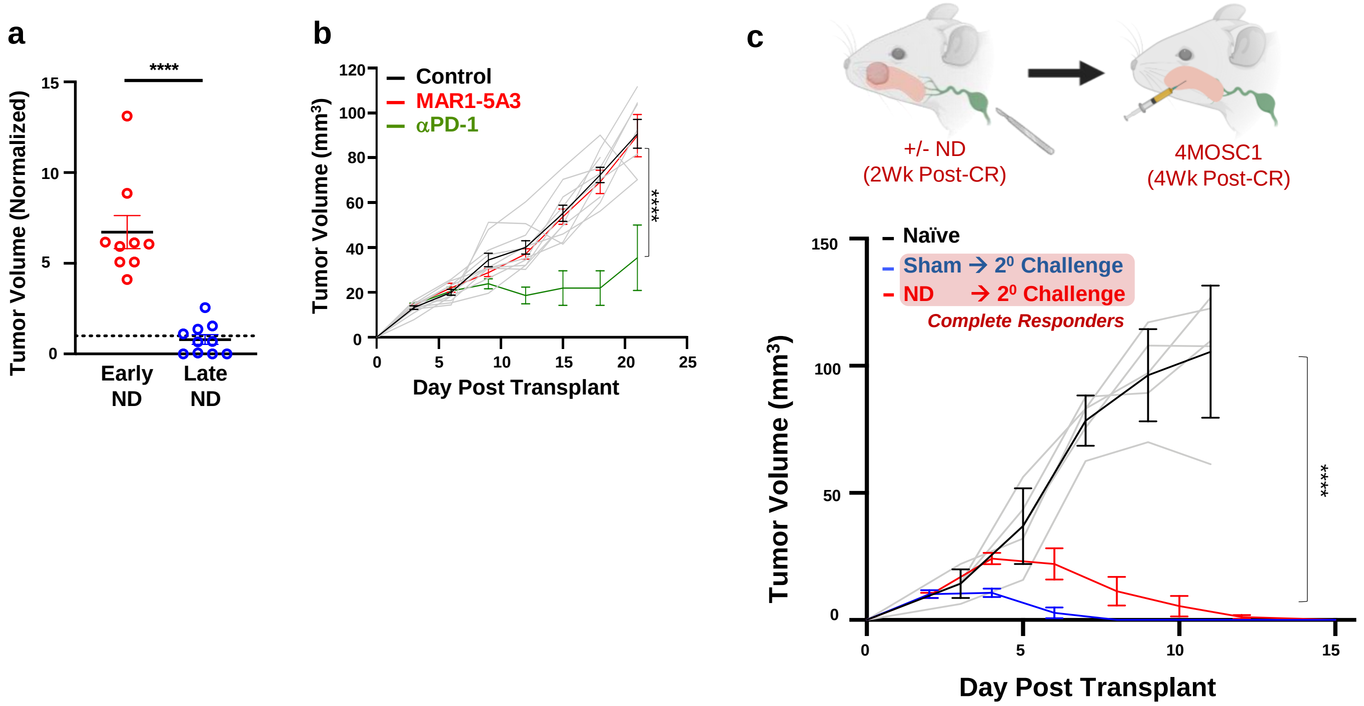

Supplementary Fig. 5: Rational IO Treatment-Sequencing Drives Primary Tumor Treatment Responses and Immunosurveillance to Protect Against Locoregional Nodal Metastasis

(A) Associated with Fig. 5B; Normalized tumor volumes comparing early (red circles, n=9) and late neck dissection (blue circles, n=10) cohorts across experiments at day 18 .

(B) Associated with Fig. 5G; tumor growth kinetics from 4MOSC1 tongue tumor bearing animals comparing control animals (black lines, n=10) to aPD-1 (green lines, n=10), MAR1-5A3 monotherapy (red lines, n=6) treated animals.

(C) **(Top)** Cartoon depicting the experimental schema. 4MOSC1 tumor-bearing animals having achieved complete response to aCTLA-4 therapy were randomized to receive either sham or neck dissection surgery two weeks after tumor clearance. Subsequently, 10^6 4MOSC1 tumor cells were orthotopically transplanted into the tongues of either naïve animals or animals two weeks status post sham surgery or neck dissection surgery (four weeks after complete response to aCTLA-4 IC therapy). **(Bottom)** Tumor growth kinetics from 4MOSC1 tongue tumor bearing treatment-naïve animals (black lines, n=5) or animals' status post complete response followed by sham surgery (blue lines, n=5) or neck dissection (red lines, n=5) .

The differences between experimental groups were analyzed using independent, two-sided student *t*-tests (Supp Fig 5A) or simple linear regression analysis (Supp Fig 5B-C). All data represent averages ± SEM, excepted where indicated. **** = $p < 0.0001$. Source data are provided as a Source Data file.
