# Supplementary material for: Lymphatic-preserving treatment sequencing with immune checkpoint inhibition unleashes cDC1-dependent antitumor immunity in HNSCC
Source: Nat Commun. 2022 Jul 25;13:4298. doi: 10.1038/s41467-022-31941-w (PMC9314425; doi:10.1038/s41467-022-31941-w)
Supplement: Supplementary file 2 — Reporting Summary [file 41467_2022_31941_MOESM2_ESM.pdf]

nature research

Corresponding author(s): J. Silvio Gutkind, Robert Saddawi-Konefka

Last updated by author(s): Jun 30, 2022

Reporting Summary

Nature Research wishes to improve the reproducibility of the work that we publish. This form provides structure for consistency and transparency in reporting. For further information on Nature Research policies, see our Editorial Policies and the Editorial Policy Checklist.

Statistics

For all statistical analyses, confirm that the following items are present in the figure legend, table legend, main text, or Methods section.

n/a	Confirmed	
☐	☒	The exact sample size (*n*) for each experimental group/condition, given as a discrete number and unit of measurement
☐	☒	A statement on whether measurements were taken from distinct samples or whether the same sample was measured repeatedly
☐	☒	The statistical test(s) used AND whether they are one- or two-sided<br>*Only common tests should be described solely by name; describe more complex techniques in the Methods section.*
☐	☒	A description of all covariates tested
☐	☒	A description of any assumptions or corrections, such as tests of normality and adjustment for multiple comparisons
☐	☒	A full description of the statistical parameters including central tendency (e.g. means) or other basic estimates (e.g. regression coefficient) AND variation (e.g. standard deviation) or associated estimates of uncertainty (e.g. confidence intervals)
☐	☒	For null hypothesis testing, the test statistic (e.g. *F*, *t*, *r*) with confidence intervals, effect sizes, degrees of freedom and *P* value noted<br>*Give P values as exact values whenever suitable.*
☒	☐	For Bayesian analysis, information on the choice of priors and Markov chain Monte Carlo settings
☐	☒	For hierarchical and complex designs, identification of the appropriate level for tests and full reporting of outcomes
☒	☐	Estimates of effect sizes (e.g. Cohen's *d*, Pearson's *r*), indicating how they were calculated

Our web collection on statistics for biologists contains articles on many of the points above.

Software and code

Policy information about availability of computer code

Data collection	Vectra imaging software (Akoya Biosciences, Vectra 3) for qcquired qpTIFF images, AID EliSpot/FluoroSpot Software V7.0
Data analysis	Qupath (v0.2.3), GraphPad software (v9), TreeStar FlowJo (v10.6.2), OMIQ data analysis software 2022 (www.omiq.ai), ImarisFileConverter (9.7.2), Imaris Software (9.6.0), In Vivo Imaging System (Living Image version 3.0)<br><br>For Analysis of RNA-sequencing Data, paired end reads were aligned using STAR v2.7.9 using default options. STAR index was created using the GRCm39 primary genome FASTA and annotation files. Resulting BAM files were sorted by name using samtools v1.7 then gene counts were quantified using HTSeq-count v0.13.5. Pairwise differential expression was calculated and PCA plots were created using DESeq2 v1.32.0.<br><br>Gene set enrichment analysis was conducted using the GSEAPreranked v7.2.4 module on the GenePattern public server, gsea-msigdb.org, with 10,000 permutations and the genes mapped and collapsed to standard mouse symbols using the MSigDB mapping file version v7.457,58. The Gene Ontology (Biological Processes) and ImmunesigDB gene set collections were used59. The ranked list of genes was created using the log2-fold change (FC) calculated by DESeq2 for the comparison of aCTLA-4-treated animals receiving either early or late neck dissection. For this analysis, genes more highly expressed in late relative to early neck dissection are at the top of the ranked list. Gene ontology (GO) analysis was performed through the GeneOntology.org website using the top significant (log2FC> 1, p-value < 0.05) upregulated genes in the samples from the late neck dissection group.

For manuscripts utilizing custom algorithms or software that are central to the research but not yet described in published literature, software must be made available to editors and reviewers. We strongly encourage code deposition in a community repository (e.g. GitHub). See the Nature Research guidelines for submitting code & software for further information.

Data

Policy information about availability of data

All manuscripts must include a data availability statement. This statement should provide the following information, where applicable:

- Accession codes, unique identifiers, or web links for publicly available datasets
- A list of figures that have associated raw data
- A description of any restrictions on data availability

The RNA sequencing data generated in this study have been deposited in the Gene Expression Omnibus database under accession code GSE197250 [https://www.ncbi.nlm.nih.gov/geo/query/acc.cgi?acc=GSE197250]. The remaining data are available within the Article, Supplementary Information or Source Data file.

Field-specific reporting

Please select the one below that is the best fit for your research. If you are not sure, read the appropriate sections before making your selection.

☒ Life sciences ☐ Behavioural & social sciences ☐ Ecological, evolutionary & environmental sciences

For a reference copy of the document with all sections, see nature.com/documents/nr-reporting-summary-flat.pdf

Life sciences study design

All studies must disclose on these points even when the disclosure is negative.

Sample size	Sample sizes were chosen based on the historical data of the variability of tumor growth and treatment response observed and determined to be adequate based on the consistency of measurable differences within and between groups. Additionally, our previous publication was used as a reference: DOI: 10.1038/s41467-019-13471-0
Data exclusions	No data were excluded from these analyses.
Replication	Every experiment was replicated at least twice with near-identical results.
Randomization	For in vivo tumor studies - based on the tumor volumes on the first day of treatment, tumor bearing mice were randomly assigned to treatment groups such that each treatment group or time point/treatment group had the same average tumor volume.<br><br>For all other experiments, animal treatment groups or samples were randomly assigned.
Blinding	Investigators were blinded to group allocation during data collection.

Reporting for specific materials, systems and methods

We require information from authors about some types of materials, experimental systems and methods used in many studies. Here, indicate whether each material, system or method listed is relevant to your study. If you are not sure if a list item applies to your research, read the appropriate section before selecting a response.

Materials & experimental systems

n/a	Involved in the study	
☐	☒	Antibodies
☐	☒	Eukaryotic cell lines
☒	☐	Palaeontology and archaeology
☐	☒	Animals and other organisms
☒	☐	Human research participants
☒	☐	Clinical data
☒	☐	Dual use research of concern

Methods

n/a	Involved in the study	
☒	☐	ChIP-seq
☐	☒	Flow cytometry
☒	☐	MRI-based neuroimaging

Antibodies

Antibodies used	Anti-PD-1 antibody (clone J43, BE0033-2), anti-CTLA4 (clone 9H10, BP0131), anti-Ly6G (clone 1A8, BE0075-1) and IFNAR depleting antibodies (Clone MAR1-5A3, BE0241) were purchased from Bio X Cell (West Lebanon, NH).<br><br>Cell surface staining was performed for 30 min at 4 °C with the following mouse antibodies (biolegend antibodies): CD45 (30-F11) (1:100), CD3 (17A2) (1:200), CD8a (53-6.7) (1:100), CD4 (RM4-4) (1:100), Slamf6 (330AJ) (1:100), PD-1 (29F.1A12) (1:100), CD44 (IM7) (1:100), CD19 (6D5) (1:100), CXCR3 (S18001A) (1:100), Tim3 (RMT3-23) (1:100), NK1.1 (PK136) (1:100), CD69 (H1.2F3) (1:100), CD62L (MEL-14) (1:100), BST2 (129C1) (1:100), Ly6C (HK1.4) (1:100), CD11b (M1/70) (1:100), CD11c (N418) (1:100), Siglec H (551) (1:100), XCR1 (ZET) (1:100), CD64 (X54-5/7.1) (1:100), CD103 (2E7) (1:100), SIRPa (P84) (1:100), MHCII (M5/114.15.2) (1:200), CD80

(16-10A1) (1:100), CD86 (GL-1) (1:100), Ep-CAM (G8.8) (1:100) and H-2Kb-SIINFEKL (25-D1.16) (1:100).
Intracellular antibodies used: IL-2 (JES6-5H4) (1:100) and IFNg(XMG1.2) (1:100).

For Cytof, Cells were stained with the following antibodies from Fluidigm: B220 (RA3-6B2), CD117(2B8), CD11c (N418), CD25 (3C7), CD4 (RM4-5), CD45 (30-F11), CD8a (53-6.7), MHC-II (M5/114.15.2), NKP46 (29A1.4), CD169 (3D6.112), CD206 (C068C2), and TCRb (H57-597); or from BioLegend: CD103 (2E7), CD115 (AFS98), CD11b (M1/70), CD19 (6D5), CD3 (145-2C11), CD64 (X54-5/7.1), F4/80 (BM8), FR4 (TH6), Ly6C (HK1.4), Ly6G (1A8), and NK1.1 (PK136); or from eBioscience (ThermoFisher Scientific): Siglec-F (1RNM44N). All antibodies were used at a 1:100 dilution.

Immunohistochemistry on formalin fixed paraffin embedded lymph node samples or tumor samples was performed using anti-wide spectrum cytokeratin antibody (Abcam, ab9377, 1:200 dilution, overnight at 4 degrees C), CD8 (Abcam ab22378, 1:400 dilution overnight at 4 degrees C) or CD4 (ab183685, 1:400 dilution overnight at 4 degrees). Tissues were then counterstained with biotinylated anti-rabbit secondary (Vector Labs, BA-1000, 1:400 dilution, 30 minutes at room temperature) or Goat Anti-Rat IgG H&L (HRP) (ab205720, 1:400, 30 minutes at room temperature).

For multiplex immunofluorescence: Rabbit anti-CD11c (D1V9Y, Cell Signaling Technology, 1:250) , anti-rabbit secondary HRP (Vector Labs, MP-7451-15), rat anti-CD8 (4SM15, ThermoFisher, 14-0808-82, 1:1750), anti-CD103 (Abcam, ab224202, 1:1500), rabbit anti-CD3 antibodies (SP7, Abcam, ab16669, 1:75),

Validation

All antibodies were validated by the supplier and were checked in the lab by comparing to the manufacturer's or in-house results.

Statement from BioLegend: BioLegend antibodies undergo an extensive series of testing to ensure quality at every step in the manufacturing process, as well as maintaining quality after the sale. Statement from Bio X Cell: Our InVivoPlus™ antibodies feature all the great qualities of our InVivoMab™ antibodies.

Statement from Bio X Cell: Our InVivoPlus™ antibodies feature all the great qualities of our InVivoMab™ antibodies. The InVivoPlus™ versions of our products are structurally and functionally identical to the InVivoMab™ versions with the added benefit of additional QC measures. InVivoPlus™ antibodies are screened for murine pathogens using ultrasensitive qPCR, screened for protein aggregation via dynamic light scattering, feature advanced binding validation via flow cytometry, ELISA, and/or Western blot, and are guaranteed to contain less than 1 endotoxin unit per milligram. Our InVivoPlus™ line of antibodies are designed to exceed the strict demands and rigorous standards required for in vivo work at any research organization.

Statement from Cell Signaling Technology: https://www.cellsignal.com/contents/our-approach-antibody-validation-principles/antibody-validation-for-immunofluorescence/ourapproach-validation-if

Statement from ThermoFisher Scientific: Thermo Fisher Scientific is committed to adopting validation standards for our Invitrogen antibody portfolio. The Advanced Verification badge is applied to products that have passed application and specificity testing. This badge can be found in the search results and at the top of the product specific webpages. Data supporting the Advanced Verification badges can be found in product specific data galleries.

Statement from Fluidigm: please see antibody information from this link: https://www.fluidigm.com/citations/antibody-validation, and antibody titration information from this link: https://www.fluidigm.com/citations/titration-of-mass-cytometry-reagents.

Statement from abcam: Antibody specificity is confirmed by looking at cells that either do or do not express the target protein within the same tissue. Initially, our scientists will review the available literature to determine the best cell lines and tissues to use for validation. We then check the protein expression by IHC/ICC to see if it has the expected cellular localization (Figure 3). If the localization of the signal is as expected, this antibody will pass and is considered suitable for use in IHC/ICC. We use a variety of methods, including staining multi-normal human tissue microarrays (TMAs), multi-tumor human TMAs, and rat or mouse TMAs during antibody development. These high-throughput arrays allow us to check many tissues at the same time, providing uniformly as all tissues are exposed to the exact same conditions.

Statement from ThermoFisher Scientific: Thermo Fisher Scientific is committed to adopting validation standards for our Invitrogen antibody portfolio. The Advanced Verification badge is applied to products that have passed application and specificity testing. This badge can be found in the search results and at the top of the product specific webpages. Data supporting the Advanced Verification badges can be found in product specific data galleries.

Eukaryotic cell lines

Policy information about cell lines

Cell line source(s)

4MSOC1 syngeneic cell lines were generated in-house and manipulated according to our previous publicatoin: DOI: 10.1038/s41467-019-13471-0. MOC1 cells were generously provided by Dr. R. Uppaluri lab. 293T cells (ATCC CRL-3216) were cultured in Dulbecco's Modified Eagle's Medium (DMEM) supplemented with 10% Fetal Bovine Serum, 2mM L-glutamine (ATCC 30-2214) and 1% antibiotic/ antimycotic solution.

Authentication

DNA authentication of cell lines was confirmed by multiplex STR profiling (Genetica DNA Laboratories, Inc. Burlington, NC) to ensure the consistency of cell identity.

Mycoplasma contamination

All cell lines are frequently tested for mycoplasma contamination. No presence of mycoplasma was found according to Mycoplasma Detection Kit-QuickTest from Biomake (Houston, TX, USA).

Commonly misidentified lines (See ICLAC register)	No commonly misidentified cells were used.

Animals and other organisms

Policy information about studies involving animals; ARRIVE guidelines recommended for reporting animal research

Laboratory animals	All the animal studies using oral cancer modelling studies were approved by the Institutional Animal Care and Use Committee (IACUC) of University of California, San Diego, with protocol ASP #S15195. Animal housing conditions are described in the manuscript.<br><br>WT C57Bl/6 mice were obtained from Charles River Laboratories (Worcester, MA). C57Bl/6 OT-1 (Tg-TcraTcrb-1100Mjb/J), IFNAR KO (Ifnar1tm1.2Ees/J) and BATF3 KO (Batf3tm1Kmm/J) animals were obtained from The Jackson Laboratory (Bar Harbor, ME). C57Bl/6 XCR1DTRVenus animals were a kind gift from Dr. Tsuneyasu Kaisho (Wakayama Medical University). Depletion of DTRvenus-expressing cells was achieved with intraperitoneal injection of diptheria toxin (DT 322326, Millipore Sigma) at a dose of 25ng/g body weight every three days. All animals used for primary tumor studies were 6-8 weeks of age and weighing 18-20g. Memory experiments made use of animals up to and beyond 52 weeks of age.
Wild animals	Study did not involve wild animals.
Field-collected samples	Study did not involve samples from the field.
Ethics oversight	All studies were approved by the Institutional Animal Care and Use Committee (IACUC) of University of California, San Diego, and mouse procedures were performed following ACP guidelines.

Note that full information on the approval of the study protocol must also be provided in the manuscript.

Flow Cytometry

Plots

Confirm that:

- [x] The axis labels state the marker and fluorochrome used (e.g. CD4-FITC).
- [x] The axis scales are clearly visible. Include numbers along axes only for bottom left plot of group (a 'group' is an analysis of identical markers).
- [x] All plots are contour plots with outliers or pseudocolor plots.
- [x] A numerical value for number of cells or percentage (with statistics) is provided.

Methodology

Sample preparation	Tumors were isolated, minced, and re-suspended into the Tumor Dissociation Kit (Miltenyi Biotec, San Diego CA) diluted into DMEM for subsequent processing with the gentleMACS Octo Dissociator, according to manufacturer's recommendations for tumor dissociation into single cell suspension. Digested tissues were then passed through a 70-µm strainers to produce a single- cell suspension. Samples were washed with PBS and processed for live/dead cell discrimination using Zombie viability stains (Biolegend, San Diego CA). Cell suspensions were then washed with cell staining buffer (Biolegend 420201) prior to cell surface staining, performed at the indicated antibody dilutions for 30 min at 4°C and protected from light. Stained cells were washed and then fixed with BD cytofix for 20 minutes at 4°C, protected from light. In the case of intracellular staining, permeabilization was then performed by incubating with fixation- permeabilization buffer (ThermoFisher 88-8824-00) according to manufacturer's recommendations prior to staining with intracellular targeted antibodies at the indicated dilutions in permeabilization buffer for 30 minutes at 4°C and protected from light. Cells were washed twice with permeabilization buffer and subsequently with cell staining buffer.
Instrument	BD LSRIFortessa
Software	TreeStar FlowJo, version 10.6.2
Cell population abundance	Transduced 4MOSC1 cells were sorted by FACS for viability and GFP-positivity using a FACS-Aria Cell Sorter (BD Biosciences), taking only the top 5% of viable GFP+ cells as the post-sort population.
Gating strategy	Single cells were gated from FSC/SSC (height/width) and live/dead cells were discriminated using Biolegend Zombie Fixable Viability Stains - BV510 Aqua, BV421 Violet, APC-Cy7 NIR or BUV395 UV. For each experiment, cells were further characterized and gated as detailed in the extended figure strategies. In general, compensation and voltages were set so as to define + populations as those > 10^3 and negative populations as those < 10^3.

- [x] Tick this box to confirm that a figure exemplifying the gating strategy is provided in the Supplementary Information.
